# Dynamic Role of Exosome microRNAs in Cancer Cell Signaling and Their Emerging Role as Noninvasive Biomarkers

**DOI:** 10.3390/biology12050710

**Published:** 2023-05-12

**Authors:** Jaya Aseervatham

**Affiliations:** Department of Neurology, Thomas Jefferson University, Philadelphia, PA 19107, USA; jaya.aseervatham@jefferson.edu or jayaa2020@gmail.com

**Keywords:** extracellular vesicles, exosomes, exosomal miRNA, noninvasive biomarkers, ESCRT, Rab GTPases, breast cancer, liver cancer, prostate cancer, colorectal cancer

## Abstract

**Simple Summary:**

Exosomes are vesicles that vary between 40 and 150 nm in diameter and are secreted by cells. These carry miRNAs that have various roles in intercellular signaling. They have different biological functions and are unique to the secreting cell. This provides information about the state of the cell and its involvement in pathological processes such as cancer. Exosomes play an important role in cancer signaling since they have both pro- and antitumor functions. In addition, they can render cells sensitive or resistant to chemotherapeutic agents. Exosomal miRNAs play an important role in promoting tumor growth and metastasis by altering the signaling pathway and promoting angiogenesis. They increase the metastatic ability of cancer cells by increasing the secretion of matrix metalloproteinases, which degrade the extracellular matrix, thereby allowing cells to spread to different parts of the body. The level of miRNAs in exosomes can be used to identify the cancer stage and grade, which eliminates the need for invasive cancer biopsies. Since exosomes can cross the blood–brain barrier, they are an excellent choice for drug delivery. As exosomes are isolated from the cells or biological fluids of the patient, their use minimizes the risk of infection and inflammation. A better understanding of exosomes and their cargoes can be exploited in the development of cancer therapeutics.

**Abstract:**

Exosomes are extracellular vesicles that originate from endosomes and are released by all cells irrespective of their origin or type. They play an important role in cell communication and can act in an autocrine, endocrine, or paracrine fashion. They are 40–150 nm in diameter and have a similar composition to the cell of origin. An exosome released by a particular cell is unique since it carries information about the state of the cell in pathological conditions such as cancer. miRNAs carried by cancer-derived exosomes play a multifaceted role by taking part in cell proliferation, invasion, metastasis, epithelial–mesenchymal transition, angiogenesis, apoptosis, and immune evasion. Depending on the type of miRNA that it carries as its cargo, it can render cells chemo- or radiosensitive or resistant and can also act as a tumor suppressor. Since the composition of exosomes is affected by the cellular state, stress, and changes in the environment, they can be used as diagnostic or prognostic biomarkers. Their unique ability to cross biological barriers makes them an excellent choice as vehicles for drug delivery. Because of their easy availability and stability, they can be used to replace cancer biopsies, which are invasive and expensive. Exosomes can also be used to follow the progression of diseases and monitor treatment strategies. A better understanding of the roles and functions of exosomal miRNA can be used to develop noninvasive, innovative, and novel treatments for cancer.

## 1. Introduction

Extracellular vesicles (EVs) are nano-sized membranous compartments made up of a lipid bilayer and transmembrane proteins that are similar in composition to the plasma membrane. They enclose proteins, nucleic acids, and other cellular components and are released by almost all cell types. Depending on the origin, they are classified as exosomes, microvesicles, or apoptotic bodies [1]. The diameter of exosomes ranges from 40 to 150 nm, and they float in a sucrose gradient at a density from 1.13 to 1.19 g/mL [2]. Although there are various isolation methods, the most frequently used is ultracentrifugation, followed by characterization by nanoparticle tracking analysis and identification by Western blot. The cargoes within the exosome are heterogeneous, often reflecting the characteristics of the cell of origin. Once packed, they are less susceptible to degradation due to their lipid bilayer and can function in an autocrine, paracrine, or even endocrine fashion [3]. Their unique property of being cell-specific can be used to relay information on the disease, and since they are able to pass through biological barriers, they have emerged as excellent candidates for drug delivery and strategizing treatment options [4]. Circulating microRNAs (miRNAs) offer a promising platform as biomarkers due to their ready availability in body fluids, tissue-specific expression, and stability. As miRNAs carried by exosomes play a crucial role in cell proliferation, invasion, metastasis, and angiogenesis, the objective of this review is to provide an overall view of the role of exosomal miRNAs in different types of cancer. The review starts with a brief introduction to exosomes, their biogenesis and secretion, and the different types of cargo, followed by a detailed review of the role of exosomal miRNA in cancer signaling pathways, alterations in the tumor microenvironment, and transforming fibroblasts into cancer-associated fibroblasts (CAFs). In addition, their role in rendering cells sensitive or resistant to chemo- or radiotherapy, their association with hormone receptor status and osteoblast modulation, and how they function as tumor suppressors are also discussed.

### 1.1. Exosome Biogenesis

**Figure 1:**
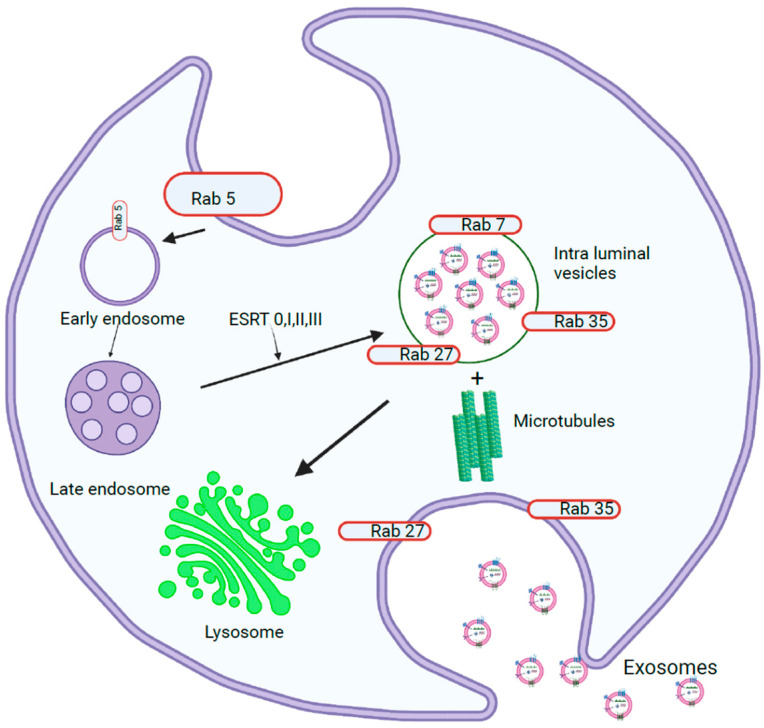
Exosome biogenesis. This figure shows the biogenesis from the endosome, its association with ESRT and Rab proteins, and its release into the extracellular environment.

Exosome biogenesis begins in the endosomal system, where early endosomes grow into late endosomes, which then form multivesicular bodies (MVBs) (Figure 1). During this process, the endosomal membrane invaginates to form intraluminal vesicles (ILVs), in which the Endosomal Sorting Complex Required for Transport (ESCRT) plays a major role [5]. The ESCRT machinery consists of four complexes, ESCRT-0, -I, -II, and -III, and their associated proteins, which have specific functions during exosome biogenesis. ESCRT-0 consists of Hrs, STAM, Vps27, and ubiquitin- and clathrin-binding domains in the amino terminus of the GAT domain. Hrs acts as a bridge that binds endosomal phosphatidylinositol 3-phosphate and ESCRT-0 to endosomal membranes [6]. The C-terminus of the Hrs subunit recruits ESCRT-I, which consists of Tsg101, Vps28, Vps37, and Mvb12. ESCRT-II is composed of one Vps36, one Vps22, and two Vps25 subunits. This binds to ESRT-I through the GLUE domain present in the carboxy terminus of Vps28. Together, ESCRT-I and ESCRT-II induce endosome budding away from the cytoplasm, during which cargoes bound to ESCRT-0 are relocated along with other cargoes [7]. ESCRT-III consists of charged subunits (Vps20, Vps32, Vps24, and Vps2) that assemble into multimers on membranes. When Vps20 binds to the Vps25 subunit of ESCRT-II, it recruits Vps20 and assembles Vps32 into filamentous oligomers capped by Vps24, which in turn recruits ATPase Vps4. This supercomplex polymerizes as a coil around the neck of the budding ILV and serves as a drawstring to the ILV pouch, promoting vesicle budding, which is stabilized by the adaptor protein ALIX. The recycling of the ESCRT-III complex from the endosomal membrane is promoted by the ATPase Vps4 complex. MVBs can be directed to lysosomes or transported to the plasma membrane, where they fuse and release ILVs into the extracellular space as exosomes, or can be directed to the plasma membrane to be loaded onto MHC II molecules or be recycled [8].

In addition to the ESCRT-dependent mechanism, exosomes can also be released through an ESCRT-independent pathway. In this pathway, cargo is sorted into specific lipid-raft-enriched microdomains that are associated with ILVs. Within the subdomains on the endosomal membrane, sphingomyelinases convert sphingomyelin to phosphorylcholine and ceramide, which induces the coalescence of these subdomains into larger domains, promoting budding and ILV formation [9]. In addition, tetraspanins (CD81, CD82, and CD9) and HSP70 recognize specific motifs in cytosolic proteins and recruit them to ILVs. A cavity filled with cholesterol is formed by CD81, which is shared by CD82 and CD9 [10]. Proteins containing KFERQ-like sequences can be sorted into exosomes by lysosome-associated membrane protein 2 isoform A, which relies on HSC70, CD63, Alix, Syntenin-1, Rab31, and ceramides [11].

#### 1.1.1. miRNA Sorting and Release of Exosomes

**Figure 2:**
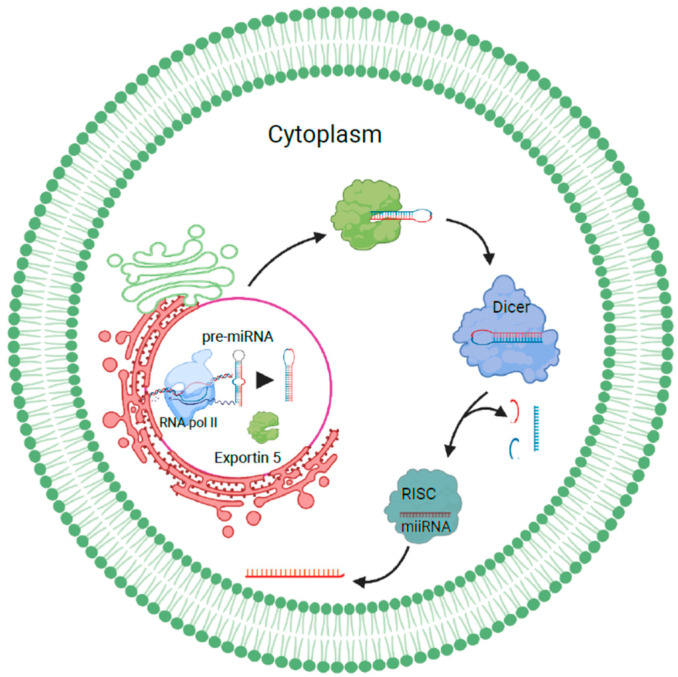
miRNA sorting in exosomes. This figure shows how miRNAs are sorted within the exosomes and how proteins help during the process.

miRNAs transcribed as long pri-miRNAs by RNA polymerase II are cleaved by Drosha-Pasha/DGCR8 in the nucleus to release hairpin-shaped precursor miRNAs. These are then exported to the cytoplasm and processed by Exportin 5. In the cytoplasm, they are cleaved by dicer into miRNA duplexes. One of the strands is then transferred to the RNA-induced silencing complex, which mediates the cleavage or silencing of target RNAs. miRNA sorting can take place by the neural sphingomyelinase 2-dependent pathway, sumoylated hnRNP-dependent pathway, 3′ miRNA sequence-dependent pathway, or the AGO2/miRISC-related pathway [12]. Sumoylated hnRNPA2B1 sorts miRNAs by recognizing the GGAG motif in their 3′ portions, while the 3′ ends of uridylated endogenous miRNAs can be used as a sorting signal as well. AGO2, which is a component of the miRISC pathway, binds to either U or A at the 5′ end of miRNAs and plays an important role in mediating mRNA–miRNA interactions [13]. Sorting can also be facilitated by the phospholipid bilayer, where specific RNA sequences are favored when they interact with lipid rafts. Once sorted, these miRNAs can be trafficked into recipient cells, where they interact with signaling receptors or directly fuse with the plasma membrane to release their contents, or they can be internalized by the recipient cell, where they fuse with endosomes or lysosomes to undergo degradation [14] (Figure 2).

#### 1.1.2. Role of Heterogeneous Nuclear Ribonucleoproteins in Sorting RNAs into Exosomes

RNA-binding proteins play an important role in sorting RNAs into exosomes. Heterogeneous nuclear ribonucleoproteins (hnRNPs) (hnRNPA2B1, hnRNPC1, hnRNPG, hnRNPH1, hnRNPK, and hnRNPQ) with different RNA-binding capacities have been associated with exosome RNA maturation, transport, stability, and translation. They have four unique RNA-binding domains, namely, an RNA recognition motif, a quasi-RRM, an RGG box, and a KH domain, which help in homologous and heterologous interactions with other hnRNPs [15]. YBX1, found in exosomes, colocalizes with cytoplasmic P-bodies containing members of the RISC complex and may undergo dephosphorylation at T271 to reach exosomes. This is required to sort tRNAs, YRNAs, and vault RNAs [16]. AGO2, along with the GW182 protein, localizes to endosomes and MVBs, suggesting that miRNAs may be mediated by the RISC complex. MEX3C colocalizes with AP-2, which is a cargo adaptor in clathrin-mediated endocytosis [17].

#### 1.1.3. Exosome Release into Extracellular Space

Exosome release into the extracellular space is facilitated by the fusion of the MVB membrane with the plasma membrane, which can be Rab GTPase (RAB11 and RAB35, or RAB27A and RAB27B)-dependent or Rab GTPase-independent. Rab GTPases recognize tether proteins on acceptor membranes and bind to them [18]. The association of the vesicle (v)-SNARE and target (t)-SNARE bring membranes into proximity, while Rab27 and Rab35 dock the MVB to the plasma membrane. After docking, Rab11 causes membrane abscission. Exosomes can also be released by Rab-independent mechanisms, such as clathrin-mediated, caveolin-mediated, or lipid-raft-mediated endocytosis, macropinocytosis, and phagocytosis. The pathway that a cell adopts depends on the cell type, stimulus, or cargo, confirming that different pathways are utilized by the cell for exosomes [19].

#### 1.1.4. Exosome Cargoes

The characterization of EV and exosome contents has been extensively documented, which has led to the creation of databases such as EVpedia and Vesiclepedia [20]. Although the results show that they are heterogeneous and change in response to external stimuli, they contain RNA, DNA, and proteins from the cell of origin. The hallmark proteins include tetraspanins (CD9, CD63, and CD81), heat-shock proteins (Hsp90), and components of ESCRT such as TSG101 and ALIX, all of which are used as markers to confirm the presence of exosomes [21]. Exosomes also contain conserved proteins and inflammation mediators [22]. Many of these proteins are present as peripheral proteins bound to the surface, which could be used as indicators of the biological state. In addition, connexins such as Cx26 and Cx43, present as integral membrane proteins, are studied for their role in cancer and other diseases [23].

The lipids present in exosomes include sphingomyelin, phosphatidyl serine, diacylglycerol, triacylglycerol, cholesterol, glycerophospholipids, glycerolipids, sterols, ganglioside GM3, and ceramide or its derivatives and hexosylceramide, which are reminiscent of lipid rafts in the plasma membrane [24]. Lipids differ in content and composition according to the cell of origin, where some may be present in greater quantities than others. Many of these have been used as biomarkers in lipid-associated diseases such as multiple sclerosis or Niemann–Pick type C disease, as altered lipid composition is present in patients with these conditions [25].

**Figure 3:**
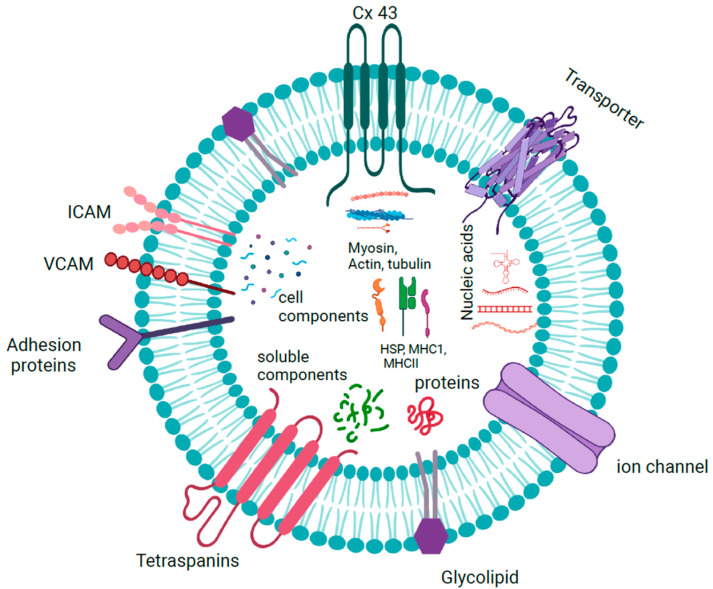
The structure of an exosome showing the lipid bilayer, its associated proteins, lipids, and cellular components.

Exosomes have been shown to contain both RNA and DNA as their cargoes (Figure 3). Double-stranded DNA was found in almost all exosomes isolated from tumor cells irrespective of their origin, which included mutations and transposable elements from their cellular origin [26]. Almost all types of RNAs, including mRNAs, tRNAs, snoRNAs, miRNAs, hnRNAs, lncRNAs, mtRNAs, and circular RNAs, are found in exosomes. These exosomal RNAs are functional and translated into proteins in the target cells, where they inhibit or enhance the expression of the target genes [27].

#### 1.1.5. Exosomes in Cancer

Exosomes, as mediators of intercellular communication, have received much attention in cancer biology due to their ability to modify the tumor microenvironment. They have been implicated in cancer metastasis, as they play a role in preparing the premetastatic niche by mediating miRNA transfer [28]. Exosomes bear the signature and reflect the RNA and protein composition of the host cell, which is unique to that cell. They contain bioactive compounds and metabolites from the secreted cell that can activate/suppress RNA or protein expression in the recipient cells by modulating signal pathways related to tumor progression, metastasis, EMT, and apoptosis. They also take part in radio- or chemoresistance, rendering the tumor cell indestructible [29].

### 1.2. Liver Cancer

Liver cancer accounts for more than 700,000 deaths each year, ranking fourth in terms of mortality, and includes hepatocellular carcinoma (HCC) and intrahepatic cholangiocarcinoma. The main risk factors are infection with hepatitis B or C virus, aflatoxin-contaminated foods, and smoking. The 5-year survival rate is about 20% due to the delayed diagnosis of HCC, recurrence, and a poor prognosis [30]. Early diagnosis is pivotal in increasing the survival rate, and miRNAs can play a major diagnostic and prognostic role in HCC due to their high specificity and sensitivity.

#### 1.2.1. Exosomal miRNA Signaling in Liver Cancer

Serum exosomal miR-665 can be used as a biomarker for HCC diagnosis and prognosis. HCC patients with higher miR-665 levels had large tumors with advanced stages, accompanied by local invasion and metastasis. Patient survival was directly proportional to the level of exosomal miR-665. In vitro and in vivo experiments confirmed that miR-665 upregulates the MAPK/ERK pathway to increase tumorigenesis [31]. Exosomes isolated from HCC patient samples showed the overexpression of miR-10b-5p, miR-18a-5p, miR-215-5p, and miR-940, which were associated with a poor prognosis. Validation by qRT-PCR revealed serum exo-miR-10b-5p as a promising biomarker for early-stage HCC, while serum exo-miR-215-5p can be used as a prognostic biomarker, confirming the fact that the differential expression of miRNAs can be used to differentiate the early and late stages of HCC [32]. ADAM10, a metalloprotease, plays a vital role in HCC. Its relationship with miR-451a was studied in HCC and adjacent normal tissues by Xu et al., who found an inverse relation between the two. The low expression of miR-451a and high expression of ADAM10 indicated the poor prognosis of HCC patients. Exosomes extracted from hucMSCs and cocultured with Hep3B and SMMC-7721 cell lines showed decreased expression of miR-451a and increased expression of ADAM10. The overexpression of miR-451a or the inhibition of ADAM10 rendered cells sensitive to paclitaxel resistance, decreased cell proliferation, invasion, and EMT, and increased apoptosis [33]. Exosomal miRNA expression can be used to distinguish between patients with liver cirrhosis and HCC. miR-16 levels were decreased in cirrhosis patients and differed among tumor stages, and exosomal miR-192 was associated with overall survival. The expression of exosomal miR-146a and miR-221 was significantly increased in HCC patients when compared to liver cirrhosis patients [34]. HCC-cell-derived exosomes were positively correlated with the activation of hepatic stellate cells (HSCs). Cell growth was dose-dependent, and the number of cells increased in the S phase, leading to increased cell proliferation. It also increased the expression of miRNA-21, which reduced the phosphorylation of PTEN and PDK1 and increased the phosphorylation of AKT, which confirms the involvement of the PTEN/PDK1/AKT pathway in the activation of HSCs [35]. Exosomal miR-93 could influence HCC carcinogenesis by promoting cancer cell proliferation and invasion. The pathway analysis of cells treated with exosomal miR-93 revealed that signal transduction pathways such as RAS, MAPKK, JNK, and apoptosis were significantly upregulated. Proliferation, invasion, and metastasis were increased by modulating the expression of TP53INP1, TIMP2, and CDKN1A. In patients, increased serum exosomal miR-93 was correlated with large tumor size and stage III or IV cancer, and these patients had poor prognoses and overall survival [36]. Exosomes from HCC cells enriched with miR-452-5p were able to induce macrophage polarization and increase invasion and migration. They also increased the expression of macrophage markers such as CD206, IL-10, and Arg-1. In vivo results showed that miR-452-5p exerted this effect by targeting TIMP3. HCC patients with high expression of miR-452-5p had poorer survival rates than those with lower expression [37].

Exosomes play an important role in tumor self-seeding in HCC, which increases metastasis and leads to aggressive cancer. The transfer of miR-25-5p by tumor-derived exosomes increased transendothelial migration and anoikis-resistant HCC by regulating the expression of LRRC7. In vivo results showed that miR-25-5p determines the self-seeding ability of the tumor [38]. miRNAs such as miR-638, miR-663a, miR-3648, and miR-4258 were able to attenuate endothelial junction integrity by inhibiting VE-cadherin and ZO-1 expression. High-miR-103-expressing exosomes derived from hepatoma cells increased the permeability of human umbilical vein endothelial cell (HUVEC) endothelial monolayers. The expression of junctional proteins such as VE-cadherin, p120, and ZO-1 was decreased, leading to increased contact in endothelial cells. Mice injected with hepatoma cells expressing high levels of miR-103 had increased tumor cells in the circulation and increased hepatic and pulmonary metastasis. Decreased expression of junctional proteins was observed, mimicking the in vitro effect of miR-103 [39]. HCC patients with higher levels of exosomal miR-638 were associated with increased vascular permeability, metastasis, and tumor recurrence [40]. Exosomal miR92a-3p induced EMT in HCC by targeting PTEN and regulating downstream signaling by modulating the AKT/Snail pathway. The increased expression of E2F1 and c-Myc upregulated the expression of miR92a-3p in highly metastatic cells (97hm and Huhm cells) by binding to the promoter of miR17HG. In patients, high plasma exosomal miR-92a-3p was associated with overall survival and disease-free survival, indicating a poor prognosis [41]. Overexpression of miR-1290 in HCC increased angiogenesis by decreasing the expression of the SMEK1 protein, which inhibited VEGFR2 phosphorylation [42]. HCC cells cocultured with exosomes isolated from CSQT-2 with elevated miR-25 expression were able to increase the malignant phenotype of HCC cells by targeting the SIK1 protein and decreasing its expression. When injected into mice, they increased tumor growth and metastasized to the lung and liver and activated the Wnt/β-catenin pathway, whereas these effects were reverted when treated with a miR-25 inhibitor [43]. Exosomes released in a hypoxic environment contain microRNAs, which play an important role in HCC progression. miR-1273f was present at higher levels under hypoxic conditions and activated Wnt/β-catenin by targeting LHX6, an inhibitor of the Wnt/β-catenin pathway. miR-1273f increased the expression of EMT markers and β-catenin, along with its downstream targets c-Myc and cyclin D1, while the level of LHX6 decreased [44]. Exosomal miR-126 derived from hepatoblastoma cells promoted tumorigenesis through the differentiation of BMSCs into cancer stem cells. It increased the expression of IL-6, Oct4, SRY, TGF-β, CD44^+^ CD90^+^, and CD133^+^ in cells. The downregulation of miR-126 was able to reverse the above effects in xenograft mice [45].

miRNAs derived from exosomes from the supernatant of HCC cells cultured under normal and acidic conditions showed the differential expression of miR-21 and miR-10b, both of which were upregulated in exosomes isolated from acidic conditions. Under acidic conditions, increased HIF-1α and HIF-2α could directly bind to the HRE-containing promoter regions of miR-21 and miR-10b and upregulate their cellular expression. EMT-related proteins such as vimentin and Snail and E-cadherin levels were controlled by the expression of miR-21 and miR-10b. Serum exosomes from HCC patients showed increased levels of miR-21 and miR-10b, which were associated with poor disease-free survival [46]. In HCC, ER stress was induced by treating cells with tunicamycin, and high-throughput sequencing of exosomes revealed higher expression of miR-23a-3p, which upregulated PD-L1 expression through the PTEN and AKT pathway. Decreased CD8^+^ and IL2 and increased T-cell apoptosis were seen when T cells were cocultured with exosome-stimulated macrophages, which could be the mechanism by which tumor cells escape cell immunity [47]. Golgi membrane protein 1 (GOLM1) is overexpressed and is a serum marker of HCC. GOLM1-containing exosomes promoted cell proliferation and migration as well as activated the GSK-3β/MMP signaling cascade. These effects were suppressed by miR-145, partially by downregulating GOLM1 and the mTOR signaling pathway [48]. M2 macrophage exosomes modified by miR-660-5p-related oligonucleotides, when cocultured with HepG2 cells, increased colony formation, proliferation, and migration abilities by regulating Kruppel-like factor 3 (KLF3). Increased expression of miR-660-5p reduced KLF3 expression and increased EMT by decreasing the expression of E-cadherin and increasing the expression of N-cadherin and vimentin. The injection of exosomes in mice increased the tumor weight and volume [49].

#### 1.2.2. Role of Exosomal miRNAs in Cancer-Associated Fibroblasts in Liver Cancer

Exosomes from CAFs contain microRNAs that are involved in HCC progression. miR-150-3p levels were lower in exosomes isolated from CAFs than in those from normal fibroblasts. Overexpression of miR-150-3p in liver cancer cells significantly inhibited cell invasion. Patients with decreased expression of miR-150-3p had higher liver fibrosis and worse survival. The same was true in the serum of patients, which might be useful as a diagnostic tool for HCC patients [50]. miR-320a expression was significantly reduced in the exosomes of CAFs derived from human HCC patients. Exosomes isolated from cells overexpressing miR-320a were able to decrease cell proliferation, colony formation, migration, and invasion and increase the number of cells in the G1 phase. miR-320a directly targeted PBX3, which is associated with EMT. Overexpression of miR-320a decreased the levels of PBX3, phospho-ERK1/2, and N-cadherin and increased the expression of E-cadherin. It also reduced the levels of MMP2, which is responsible for invasion, and CDK2, a cell cycle regulator. Transferred miR-320a could function as an antitumor miRNA by targeting *PBX3* [51]. Exosomal miR-1247-3p isolated from HCC cells was able to activate β1-integrin–NF-κB signaling by targeting B4GALT3. Activated fibroblasts expressed high levels of pro-inflammatory and collagen genes, leading to inflammation. In addition, the contraction ability was highly enhanced. Intravenous administration of exosomes from highly metastatic HCC cells increased lung metastasis in vivo, which was associated with cancer-associated fibroblasts [52]. LIMA1 is an actin-binding protein involved in controlling the progression of solid tumors and was positively associated with the overall survival of HCC patients. LIMA1 binds to BMI1 and induces its destabilization. In HCC patients, decreased expression of LIMA1 leads to increased cell proliferation, metastasis, and reoccurrence. CAF-derived exosomes containing miR-20a-5p were able to reduce the expression of LIMA1, and the treatment of mice with CAF-originated exosomes facilitated tumor growth, confirming that CAFs exerted carcinogenic effects on HCC cells via the transfer of exosomes carrying miR-20a-5p [53].

#### 1.2.3. Role of Exosomal miRNAs in Chemoresistance in Liver Cancer

miR-744 has an essential role in chemoresistance in HCC. Exosomes derived from HCC patient serum and HepG2 cells had decreased levels of miR-744, which suggests that it may have an antiproliferative role. Exosomes enriched with miR-744 decreased cell proliferation and increased sensitivity to sorafenib treatment by targeting PAX2 [54]. Levels of miR-199a in HCC tissues decreased by 80%, and in cell lines, it was correlated with chemosensitivity. The delivery of miR-199a by exosomes from adipose-tissue-derived MSCs (AMSCs) into HCC cells decreased the proliferation rate, increased the number of apoptotic cells, and rendered the cells sensitive to Dox exposure. miR-199a targeted the mTOR pathway by decreasing the phosphorylation of its downstream proteins, 4EBP1 and 70S6K, all of which could be reproduced in vivo, confirming that exosomes can be used to facilitate the sensitivity of HCC cells to chemotherapeutic agents [55]. When AMSC-derived exosomes loaded with miR-122 were added to recipient HCC cells, they negatively regulated the genes involved in drug resistance, making the cells sensitive to chemotherapeutic agents. The downregulation of genes such as cyclin G1 (CCNG1), ADAM10, and insulin-like growth factor receptor 1 (IGF1R) was confirmed by Western blot, and cells were arrested in the G0/G1 phase. This pattern was observed in vivo when mice were injected with AMSC-derived exosomes loaded with miR-122. The expression of the apoptosis-related genes caspase-3 and Bax (Bcl-2 Associated X protein) was upregulated in the 122-Exo-treated group [56]. Sorafenib treatment induced the production of larger EVs and increased the expression of miRNAs in exosomes. In HCC patients, a higher survival rate was proportional to the level of miR-200c-3p, while the poor prognosis of patients was associated with high levels of miR-222-5p and miR-512-3p after sorafenib treatment. The expression of these miRNAs can bear prognostic value and contribute to treatment response [57]. Exosomes play a role in multidrug resistance in cancer by regulating the expression of miR-32-5p. Transferring exosomes isolated from a multidrug-resistant cell line (Bel/5-FU) to a sensitive cell line (Bel7402) made the latter resistant by activating the PI3K/AKT pathway and suppressing the expression of PTEN. Xenograft mice showed the activation of the PI3K/AKT pathway and increased EMT and angiogenesis upon exosome treatment [43].

#### 1.2.4. Anticancer Effects of Exosomal miRNAs in Liver Cancer

Mesenchymal-stem-cell-derived exosomes, which are widely accepted as crucial messengers for intercellular communication, modulate the macrophage phenotype to regulate the inflammatory microenvironment in the liver and repair injury. miR-148a was found to be decreased in the serum exosomes of patients with liver fibrosis. This was negatively correlated with the FIB-4 score and APRI score, reflecting the degree of liver fibrosis. miR-148a targets Kruppel-like factor 6 (KLF6) to suppress pro-inflammatory macrophages and promote anti-inflammatory macrophages by inhibiting the STAT3 pathway, which could be exploited as a potential therapeutic target for liver fibrosis [58]. Exosomes isolated from mesenchymal stem cells (MSCs) enriched with miR-15a were able to impede cell proliferation, migration, and apoptosis in Hep3B and Huh7 cells by binding to SALL4. Western blot results showed the decreased expression of MMPs and elevated caspases in the coculture system. Exosomal miR-15a decreased the tumorigenicity and metastasis of HCC tumors in vivo [59]. Exosomes isolated from bone marrow mesenchymal stem cells (BMSCs-exo) loaded with microRNA have been used as a promising therapy for human cancers. BMSCs-exo loaded with miR-205-5p and cocultured with LM3 cancer cells were able to transfer exosomes to LM3 cells, which increased the concentration of miR-205-5p. This acts as a tumor suppressor by decreasing cell proliferation, invasion, and metastasis by regulating the expression of cyclin-dependent kinase-like 3 (CDKL3). The loss of CDKL3 impaired the malignant activities of liver cancer cells in vitro and in vivo [60]. The delivery of engineered exosomes loaded with miR-26a into HepG2 cells caused wound closure and reduced cell proliferation and migration. This effect was mediated by the arrest of the cell cycle and the downregulation of the expression of CCNE2 and CDK6, which are regulators of G1/S transition [61]. Hepatic-stellate-cell-derived exosomes enriched with miR-148a-3p were able to decrease the proliferation, invasion, and migration of HCC cells. A significant difference was found in the phosphorylation status of PI3K and AKT. The decreased expression of E-cadherin and ITGA5 confirmed that miR-148a-3p can target PI3K/AKT signaling to decrease tumorigenesis. Low miR-148a-3p expression in the plasma of patients was associated with decreased overall survival [62]. Stellate-cell-derived EVs loaded with miR-335-5p are taken up by HCC cells in vitro and exert an antitumor effect. In vivo results showed that EV treatment resulted in significantly smaller tumors and reduced the expression of ki67 and cleaved caspase-3. The targets of miR-335-5p were CDC42, CSNK1G2, EIF2C2, EIF5, etc., all of which are downstream signaling pathways. The antitumor effects of miR-335-5p may be mediated by modulating the transcriptional regulation of these targets [63].

Differentially expressed miRs (DEMs) of patients with liver cirrhosis and healthy controls showed that miR-618 was downregulated in cirrhosis patients. Loading exosomes derived from MSCs with miR-618 was able to attenuate the progression of hepatic fibrosis by decreasing the expression of SMAD4 [64]. The analysis of serum exosomal miR-320a in HCC and chronic liver disease (CLD) patients and healthy volunteers revealed lower expression in HCC when compared with the other two. Decreased expression increased metastasis, and survival analysis showed that it can be used as a diagnostic marker to distinguish between patients and controls [65]. Exosomes isolated from HCC tissues and plasma identified hsa-miR-483-5p as the only differentially expressed miRNA. It was significantly downregulated in HCC and targeted CDK15 proteins, which could be used as a biomarker to differentiate between the two [65]. Serum exosomal miR-122 and miR-148a in HCC patients were downregulated and positively related to tumor differentiation and the patient survival rate. It acted by targeting the expression of PAX2 in HCC [66]. Compared to controls, serum exosomes from patients with HCC had higher levels of miR-9-3p, which targets fibroblast growth factor 5 (HBGF-5). Overexpression of miR-9-3p in vitro downregulated the RNA and protein expression of HBGF-5, leading to reduced cell viability and proliferation and the decreased expression of ERK1/2, which suggests that miR-9-3p may be a potential therapeutic target for HCC [67]. The correlation between clinical outcome and the deregulation of serum exosomal miR-320d in exosomes isolated from the serum of HCC patients was studied by Li et al. (2020). The results of the study revealed that decreased expression resulted in lymph node metastasis and decreased patient survival [68]. miR-451a, which is a tumor suppressor, was found to be downregulated in serum exosomes from HCC patients. Overexpression induced apoptosis in HCC cell lines and decreased migration, tube formation, and vascular permeability in human umbilical vein endothelial cells by targeting LPIN1. An inverse relationship was found between miR-451a and LPIN1 expression in HCC xenografts in vivo, and antitumor effects were exerted by increasing apoptosis [69]. Exosomes isolated from mast cells treated with the hepatitis C virus E2 (HCV-E2) envelope glycoprotein incubated with HepG2 cells increased the level of miRNA-490, which inhibited the proliferation, migration, and invasion of HepG2 cells. This might be due to the downregulation of EGFR, phosphorylated AKT, and ERK1/2 [70].

### 1.3. Colorectal Cancer

Colorectal cancer (CRC) is a malignant tumor that, in 50–70% of cases, arises from adenomatous polyps that progress to hyperplasia and invasive carcinoma. It is a multi-step process resulting from genetic mutations that accumulate over time. More than 1.9 million cases occur each year, which represents about 1/10 of cancer-related deaths. The survival rate is about 90% if detected at stage I, and it decreases to 10% at stage IV [71]. Although it ranks third in terms of incidence and second in mortality, effective diagnostic and prognostic markers are lacking. Presently, colonoscopy is the standard method for screening, but it is highly invasive, cumbersome, and expensive. A fecal occult blood test is also used for diagnosis, but the sensitivity is very low, which emphasizes the need for new diagnostic and prognostic methods [72]. Since CRC is mostly caused by genetic mutations, studying miRNA expression may be an effective tool for the identification of genes involved and their role in cancer, which could serve as biomarkers.

#### 1.3.1. Role of Exosomal miRNA in Cell Signaling in Colorectal Cancer

Tumor-derived exosomes can promote the interaction between tumor and tumor-associated macrophages to promote liver metastasis in CRC. Overexpression of miR-934 in CRC patients was associated with a poor prognosis. miR-106b-3p expression was found to be higher in patients with metastasis than in those without. Exosomes from high-metastatic cells were able to induce EMT in low-metastatic CRC cells by targeting the expression of deleted in liver cancer-1 [73]. The differential expression of miRNAs can be used to distinguish between CRC patients with and without liver metastasis. miR-99b-5p was expressed at a significantly higher level in CRC patients without metastasis when compared to patients with metastasis, which was particularly prominent in stage III. Higher expression was associated with increased survival, suggesting that it may help in preventing liver metastasis [74]. The expression of miRNA-10b in CRC tissues and adjacent tissues was studied by Jiang et al., who found the increased expression of miRNA-10b in CRC tissues and patients with liver metastasis. The expression was positively correlated with advanced TNM stages, venous infiltration, poor differentiation, and decreased overall survival [75]. Exosomal miR-6803-5p was significantly increased in CRC patients at the III/IV TNM stages or with lymph node metastasis as well as liver metastasis when compared to healthy subjects. Overall survival was shorter in these patients and was associated with prognosis adjusted for age, sex, TNM stage, and lymph node metastasis [76]. Exosomes derived from EMT-induced HCT-116 cells had high expression of miR-128-3p, which led to changes in proliferation, migration, invasion, and EMT by downregulating the expression of FOXO4 and activating the TGF-β/SMAD and JAK/STAT3 signaling pathways. Clinically, high expression of miR-128-3p was associated with perineural invasion, lymphovascular invasion, tumor stage, and CA19-9 content in CRC patients [77]. The injection of SW480/miR-25-3p exosomes in mice resulted in decreased expression of KLF4, KLF2, ZO-1, occludin, and claudin5 and increased expression of VEGFR2. It also promoted hepatic and pulmonary metastasis and increased vascular permeability and angiogenesis, suggesting that cancer-derived exosomes can induce premetastatic niche formation at foreign sites [78]. The expression of miR-424-5p can be used to distinguish between CRC and control samples. Increased expression of miR-424-5p was associated with stages I and II of CRC and promoted cell proliferation and metastasis by inhibiting the expression of SCN4B [79]. miR-21 was significantly upregulated in exosomes from colon cancer cells when compared to normal human colon epithelial cells. A positive correlation was found between miR-21 and MMP-2, MMP-9, and MMP-11, while a negative correlation was found for tropomyosin 1, programmed cell death protein 4, and phosphatase and tensin homolog, suggesting that miR-21 acts by downregulating its target genes [80]. The levels of miR-21, miR-29a, miR-92a, and miR-135b were higher in CRC tissues when compared to normal tissues, and in serum, the expression of miR-21 and miR-29a was proportional to the adenoma size and polyp count [81].

miR-335-5p, highly expressed in exosomes isolated from metastatic SW620 cells, promoted cell migration, invasion, and EMT by targeting RAS p21 protein activator 1 (RASA1). Cells overexpressing RASA1 were able to downregulate the expression of Ras and vimentin and increase the expression of E-cadherin, ultimately reducing the process of EMT [64]. Overexpression of exosomal miR-224-5p promoted CRC cell proliferation, migration, and invasion via targeting CMTM4, which is a tumor suppressor. It decreased the expression of CMTM4 and upregulated the expression of p-AKT and p-ERK proteins in CCD 841 CoN cells [82]. Exosomes isolated from the serum of patients with colorectal adenocarcinoma had increased levels of Let7, miR-21, miR-23, miR-29, and miR-222, which could be used to differentiate between colorectal adenocarcinoma and normal patients [83]. Exosomes from CRC cells undergoing EMT increased the level of microRNA-106b-5p in M2 macrophages by activating the PI3K/AKT/mTOR pathway, suppressing programmed cell death 4 (PDCD4). In patients, increased expression of exosomal miR-106b increased malignant progression [84]. miR-21 and miR-10 were the most important functional miRNAs in exosomes isolated from the acidic microenvironment. These were able to promote the proliferation, migration, and invasion of recipient HCC cells. In HCC patients, serum exosomal miR-21 and miR-10b increased the expression of HIF-1α and -2α, which were associated with an advanced tumor stage and were also indicators of disease-free survival [46]. Exosomes enriched in miR-210-3p isolated from hypoxic colorectal cancer cells (H-Exos) promoted cell proliferation by increasing the cells in the S phase of the cell cycle and decreased apoptosis in normoxic cells by suppressing the expression of CELF2. miR-210-3p directly bound to the 3′-UTR of CELF2 mRNA to bring about the above-mentioned effects. Xenografts treated with H-Exos promoted tumor growth in nude mice, increased Ki67 expression, and downregulated the expression of CELF2 and cleaved caspase-3 [85]. The analysis of exosomes extracted from human umbilical cord mesenchymal stem cells cocultured with LoVo cells showed the upregulation of miR-431-5p and the downregulation of PRDX1, which suppressed the malignant behavior of LoVo cells [86]. hnRNPA2B1 mediates the transfer of miR-934 into exosomes, which are then transferred into macrophages, where they induce the polarization of M2 macrophages by downregulating PTEN expression and activating the PI3K/AKT signaling pathway. Polarized macrophages induce the formation of the premetastatic niche and promote liver metastasis by activating the CXCL13/CXCR5/NFκB/p65/miR-934 positive feedback loop in CRC cells [87].

To assess the effect of miR-183-5p on angiogenesis, exosomes isolated from CRC cells were cocultured with HMEC-1. Increased expression of miR-183-5p enhanced cell proliferation, invasion, and metastasis and increased tube formation in HMEC-1 by regulating the expression of FOXO1 [88]. When transferred to endothelial cells, exosomes containing miR-21-5p from CRC cells decreased the expression of Krev interaction trapped protein 1, activated the β-catenin signaling pathway, and increased the expression of VEGFa and Ccnd1, leading to increased vascular permeability and angiogenesis [89]. Circulating exomiR-1229 levels were significantly increased in the serum exosomes of patients with CRC. This was associated with tumor size, lymphatic metastasis, TNM stage, and poor survival and was able to promote the tubulogenesis of HUVECs by targeting the expression of HIPK2 and activating the VEGEF pathway [90]. The clinical significance of exosomal miR-27b-3p in angiogenesis was assessed in CRC patients and healthy donors. The expression of miR-27b-3p was higher in CRC patients with metastasis than in controls, which correlated with lymphovascular invasion, deep tumor invasion, lymph node metastasis, tumor stage, and positive pre-operative CTC status. In vitro studies showed that it was able to attenuate the vascular barrier and increase vascular permeability in HUVECs by binding to the 3′-UTR of VE-Cad and p120 [91].

#### 1.3.2. Role of Exosomal miRNAs in Cancer-Associated Fibroblasts in Colorectal Cancer

Cancer-associated fibroblasts (CAFs) and normal fibroblasts (NFs) were isolated from cancerous tissues and matched with paracancerous tissues to study the role of the interaction between miR-93-5p, forkhead box A1 (FOXA1), and TGFB3 in radioresistance in CRC. CAFs-exo had higher expression of miR-23a-3p than miR-93-5p, which targeted FOXA1. When exosomes overexpressing miR-93-5p and FOXA1 were cocultured with SW480 cells, exposure to irradiation induced cell cycle arrest and apoptosis and increased the number of cells in the G1/S phase, suggesting that exosomal miR-93-5p facilitated the chemoresistance of CRC cells by downregulating FOXA1 and preventing the translocation of TGFB3 into the nucleus. In vivo results showed the downregulation of BAX and the upregulation of Bcl2 [92]. When CAF-derived exosomes cocultured with CRC cells were exposed to radiation, exosomes overexpressing miR-590-3p increased cell survival by altering the ratios of p-PI3K/PI3K and p-AKT/AKT, increasing CLCA4, and decreasing the expression of cleaved PARP, cleaved caspase-3, and γH2AX in cells [93]. Exosomes extracted and purified from CAF-conditioned medium were enriched with miR-625-3p, which was associated with proliferation, migration, invasion, EMT, and drug resistance. A xenograft model with increased expression of miR-625-3p showed extensive necrosis and apoptotic nuclear fragmentation and was chemoresistant to oxaliplatin [94]. The higher levels of miR-10b expression in CRC cells than in normal colorectal epithelial cells can be used to distinguish between the two. Coculturing fibroblasts with exosomes containing miR-10b suppressed PI3K expression and inhibited the PI3K/Akt/mTOR pathway and reduced fibroblast proliferation but promoted the expression of TGFβ and smooth muscle α-actin, which suggests that miRNA-10b can activate fibroblasts to become CAFs [95]. Exosomal miR-17-5p can be transferred from parental CAFs to CRC cells to influence the metastasis of CRC. It bound to the 3’-UTR of RUNX3 and interacted with MYC. This complex then bound to the promoter of TGF-β1 and activated the TGF-β signaling pathway. A positive feedback loop is formed when TGF-β1 activates CAFs to produce more miR-17-5p, further increasing CRC progression [96]. RNA sequencing revealed the increased expression of miR-146a-5p and miR-155-5p in CXCR7-overexpressing CRC cells and their exosomes. These miRNAs could be taken up by CAFs and promote their activation by activating JAK2-STAT3/NF-κB signaling and suppressing cytokine signaling 1, zinc finger, and BTB domain containing 2. They also increased the expression of IL-6, TNFα, TGFβ, and CXCL12, leading to EMT and increased metastasis in a xenograft model [97].

#### 1.3.3. Role of Exosomal miRNAs in Chemoresistance in Colorectal Cancer

Circulating exosomal miRNAs can be used as biomarkers to determine resistance to oxaliplatin. Plasma exosomal miRNAs from oxaliplatin-sensitive and -resistant patients analyzed by microarray showed the downregulation of 20 miRNAs and the upregulation of 4 miRNAs in chemoresistant patients. miR-184, miR-100, miR-10a, and miR-92a were upregulated in the resistant group, while let-7i, miR-144, miR-30e, and miR-16 were downregulated. Pathway analysis showed that miRNAs related to transcription and signaling pathways were mostly deregulated [98]. Exosomes isolated from oxaliplatin- or 5-fluorouracil-resistant CRC cell lines showed the upregulation of miR-21-5p, miR-1246, miR-1229-5p, miR-135b, miR-425, and miR-96-5p. Serum exosomes from chemoresistant patients confirmed the upregulation of miR-21-5p, miR-1246, miR-1229-5p, and miR-96-5p when compared to sensitive patients. These miRNAs were related to PI3K-AKT signaling, FOXO signaling, and the autophagy pathway [99]. miR-46146 transferred by exosomes from oxaliplatin (OX)-resistant cells made OX-sensitive cells resistant to OX treatment by targeting PDCD10 [100]. When cocultured with oxaliplatin-resistant CRC cells, exosomes containing miRNA-1915-3p isolated from a non-tumorigenic intestinal cell line (FHC) made the latter sensitive to oxaliplatin and downregulated the EMT-promoting genes 6-phosphofructo-2-kinase/fructose-2,6-biphosphatase 3 and ubiquitin carboxyl-terminal hydrolase 2 [101]. miR-31-5p increased resistance to oxaliplatin treatment by binding to large tumor suppressor kinase 2. The expression of Forkhead box C1 (FOXC1) was directly correlated with the increased expression of miR-31-5p. FOXC1 bound to the promotor of miR-31 and upregulated the expression of miR31-5p and LATS2, conferring resistance to oxaliplatin treatment [102].

#### 1.3.4. Anticancer Role of Exosomal miRNAs in Colorectal Cancer

Exosomal miR-548c-5p from CRC cells was capable of inhibiting CRC cell proliferation, migration, and invasion by targeting the HIF1α/CDC42 axis and downregulating its expression. CRC patients with low miR-548c-5p in exosomes had a lower survival rate and poor disease-free survival [103]. Exosomal miR-34a reduced the tumor volume and tumor weight and increased survival in mice. Mice treated with tumor-derived exosomes had increased tumor necrosis and apoptosis and decreased metastasis when compared to the control group. Moreover, the expression of IL-6R, STAT3, and PAI-1 transcripts was also reduced. In addition, the level of CD4+ T cells decreased and CD8+ T cells increased in the miRNA group [104]. Mesenchymal stem cells (MSCs) enriched with miR-100 and miR-143 were able to suppress cell proliferation and induce apoptosis in CRC cells by decreasing the expression of MMPs (MMP2 and MMP9) and EMT genes (SNAIL, TWIST, vimentin, and N-cadherin). In addition, they also effectively downregulated the expression of mTOR, cyclin D1, K-RAS, and HK2 while upregulating the expression of E-cadherin and p27 [105]. Exosomal miR-150 was downregulated in CRC patients with liver metastases when compared to those without. This was associated with advanced tumor node metastasis staging, higher CA199 levels, liver metastasis, and overall survival. miR-150 directly targets FTO (the fat mass and obesity-associated gene) to decrease liver metastasis, suggesting that exosomes play an important role in liver metastasis in colorectal cancer patients [106]. Reduced exosomal miR-548c-5p was found in CRC patients with liver metastasis and at later stages when compared to controls. It was independently associated with shorter overall survival, metastasis, and vascular infiltration [107]. The expression of miR-132 was decreased in liver metastasis patients when compared to controls, which could be used as an independent prognostic factor. In vitro studies showed that the overexpression of miR-132 inhibited cell proliferation and cell invasion in CRC cell lines. A luciferase reporter revealed that anoctamin 1 (ANO1) was a direct target of miR-132 [108]. The plasma exosomal miR-140-3p level was lower in CRC patients than in healthy controls, and decreased levels were observed in CRC patients with liver metastasis when compared to matched normal tissues. Overexpression of miR-140-3p in LoVo cells suppressed cell proliferation, migration, invasion, and the nuclear translocation of β-catenin by modulating the expression of BCL9 and BCL2. Overexpression of miR-140-3p in vivo inhibited tumor growth in a LoVo xenograft model and diminished metastatic nodules in nude mouse liver and suppressed CRC progression by targeting BCL9 and BCL2 [109]. 

Serum exosomal miR-874 levels can be used to distinguish between CRC patients, benign adenomas (AD) cases, and healthy controls. The level was significantly downregulated in CRC patients than in adenomas or healthy controls. Low levels were associated with positive distant metastasis, positive lymph node metastasis, poor differentiation, and an advanced TNM stage. Moreover, it could be used as an independent prognostic factor for the overall survival of CRC patients [110]. When MSC exosomal microRNAs in CRC tissues were analyzed for the expression of miR-3940-5p, the results showed that increased expression inhibited EMT and suppressed tumor growth in vivo. When exosomes loaded with miR-3940-5p were injected into nude mice, the number of metastatic nodules in the lung was reduced when compared to control mice. In vitro studies showed that miR-3940-5p bound to ITGA6 mRNA and decreased the expression of the TGF-β1 signaling pathway [111]. Tumor and adjacent healthy tissues obtained from 100 CRC patients revealed that the expression miR-466 was significantly decreased in tumor tissues when compared to adjacent non-tumor tissues, which was associated with tumor size, node, and metastasis. Multivariate analysis revealed that low expression was associated with a poor prognosis in CRC patients. Ectopic expression in SW-620 cells induced G0/G1 arrest and apoptosis. The antitumor effects were mediated by inhibiting the expression of cyclin D1 and MMP-2 [112]. CRC tissues had lower levels of miR-506-3p in exosomes than non-tumor tissues. Increased expression of miR-506-3p negatively regulated GSTP1 to bring about antitumor effects in CRC [113]. In CRC samples, the expression of exosomal miR-150-5p was reduced, which was correlated with metastasis and the tumor stage [114].

### 1.4. Breast Cancer

Breast cancer is the leading cause of death among cancers in women and currently contributes to one-third of cancer diagnoses in women. The 5-year survival rate is 100% if diagnosed at stage I but decreases to 28% for those in stage IV [115]. Breast cancer arises from the lining of the epithelium of the ducts or lobules. Depending on the size of the tumor, it is classified as stages I-IV. Over time, regional or distant metastasis occurs depending on the severity of the disease. Currently, mammography is the standard technique for screening, but it may be overshadowed by false-positive results, which leads to additional imaging or needle biopsy [30]. Serum biomarkers such as CA 15-3, CA 27.29, and CEA help in the diagnosis of metastatic breast cancer, but they are not recommended for diagnosis or screening due to their low sensitivity. Therefore, there is a crucial need for noninvasive biomarkers that will aid in the early detection of breast cancer. Circulating microRNAs offer a promising platform due to their ready availability in body fluids, tissue-specific expression, stability, and ease of quantification. miRNAs can primarily function as tumor suppressors or tumor enhancers depending on the tissue of origin and the stage of cancer [116].

#### 1.4.1. Role of Exosomal miRNAs in Signaling Pathways in Breast Cancer

Exosomal miRNAs can increase tumorigenesis by increasing the expression of proteins involved in cell proliferation, invasion, and metastasis. Exosomes isolated from breast cancer cells with high metastatic ability (HM) significantly enhanced tube-forming ability and outgrowth or arterial rings and enhanced vascular generation in endothelial cells when compared to exosomes isolated from cells with low metastatic potential (LM). HM-Exos increased the expression of EPHA2 by activating the HIF1-α pathway through Ephrin A1-EPHA2 forward signaling [72]. Increased expression of miRNA-21-5p and miRNA-10b-5px was found in exosomes isolated from patients with grade I, II, or III breast cancer. miRNA-21-5p expression was higher in patients with tumors larger than 2.5 cm and in the Her2-positive group than in the negative one, suggesting that these miRNAs can be used as biomarkers [117]. Serum exosomal miR-148a levels started gradually decreasing when benign breast tumors transitioned to breast cancer, and they were upregulated following surgery and dropped with disease relapse. Its expression was associated with the TNM stage, differentiation, and lymph node metastasis, and patients had worse overall survival, indicating that it could be used as a biomarker [118]. Serum exosomes isolated from eighty-three breast cancer (BC) and thirty-four healthy women showed reduced levels of exosomal miR-17-5p in breast cancer patients, which could distinguish between control and breast cancer samples with higher sensitivity and specificity than serum biomarkers such as CEA, CA125, and CA153 [119]. Exosomes isolated from breast cancer patients revealed the upregulation of miR-338-3p and 124-3p and the downregulation of miR-29b-3p, miR-20b-5p, miR-17-5p, miR-130a-3p, miR-18a-5p, miR-195-5p, miR-486-5p, and miR-93-5p in patients with recurrence. miR-340 was highly expressed at the primary tumor site and as well as in serum, whereas miR-195-5p, miR-17-5p, miR-93-5p, and miR-130a-3p were downregulated in the serum of patients with recurrence compared with those without and could be used as biomarkers to distinguish between the two [120]. miR7641 was differentially expressed in metastatic cancer cells and could promote cell proliferation and invasion. When xenograft tumors were treated with exosomes with high expression of miR-7641, mice had high tumorigenicity. Breast cancer patients with distant metastasis had significantly higher levels of miR-7641 than patients without metastasis, which was correlated with disease-free survival [121].

Exosomal miRNA profiles from the peripheral blood of BC patients and age-matched healthy women revealed that miR-363-5p was significantly higher in breast cancer patients. It was decreased in lymph-node-positive patients when compared with lymph-node-negative patients. Multivariate survival analysis showed that high expression levels of miR-363 could serve as a diagnostic marker. Overexpression of miR-363-5p significantly suppressed the proliferation, migration, invasion, and colony formation of MCF-7 cells. Functional assays demonstrated that exosomal miR-363-5p targets PDGFB to inhibit cell proliferation and migration [122]. The level of plasma exosomal hsa-miR-21-5p was upregulated in breast cancer cells, tissues, and exosomes, while it was downregulated in breast cancer patients after tumor resection. miR-21-5p exerts its effect by downregulating the expression of TGFβR3 and EGFR. ROC curve analysis showed that plasma exosomal hsa-miR-21-5p was able to distinguish between healthy and breast cancer samples with high sensitivity and specificity [123]. The analysis of exosomal samples from human BC cells (MCF7 and MDA-MB-231) and normal mammary epithelial cell lines (MCF10, MCF-10A) showed the differential expression of miR-455-5p and miR-1255a. miR-455-5p regulated the expression of CDKN1B to influence the cell cycle process, and miR-1255a interacted with SMAD4 to participate in the TGF-β signaling pathway. High expression of both miRNAs was associated with poor overall survival [124]. The diagnostic value of exosomal miR-1910-3p in breast cancer was studied by Wang et al. (2020). The expression levels of miR-1910-3p were higher in MCF7 and MDA-MB-231 when compared to normal MCF-10A cells, which promoted the upregulation of N-cadherin, vimentin, slug, twist, Bcl2, PCNA, and β-catenin. It downregulated E-cadherin, cleaved caspase-3, and PARP. In vivo results showed that the tumor weight and volume were significantly increased in 1910 mimics than in controls. Immunohistochemical analysis showed increased staining intensity for N-cadherin, vimentin, and PCNA. Western blotting showed the upregulation of p-p65 and β-catenin and the downregulation of p-IκBα. In addition, the autophagy-related genes ATG7 and BECN1 and the autophagy-related protein LC3B were increased in the 1910 mimic group. In patients, exosomal miR-1910-3p was significantly higher in the serum of breast cancer patients than in that of healthy volunteers and acted by downregulating MTMR3 and activating the NF-κB signaling pathway [125]. Exosomal miR-4443 promotes the metastasis of BCa by downregulating the expression of TIMP2 and upregulating MMPs. In vivo, exosomal miR-4443 downregulated TIMP2, upregulated MMPs, and accumulated in the primary tumor [126].

Exosomes derived from high-metastatic breast cancer cells showed that miR-760 targeted the Src/PI3K/AKT signaling pathway. The expression of ARF6, p-AMAP1, vimentin, p-Src, p-PI3K, and p-AKT was upregulated, while the expression of E-cadherin was downregulated in Exo-CCL18 and miR-760 mimic groups. Knocking down ARF6 reversed the above effects, suggesting that the Src/PI3K/AKT signaling pathway is involved [127]. The analysis of miRNAs in two different breast cancer cell lines, Hs578Ts(i)_8_, the aggressive phenotype, and its isogenic colony Hs578T cells, which are less aggressive, revealed that 83 miRNAs were downregulated in both cell lines when compared to their normal Hs578T counterparts. Ten of the most downregulated miRNAs were clustered on chromosome 14, 14q32, the region that is deleted during cancer progression. miR-134 overexpression downregulated STAT5B, Hsp90, and Bcl-2 expression. This led to reduced aggressiveness, proliferation, migration, invasion, and sensitivity to cisplatin treatment [128]. miR-1246 was highly expressed in exosomes isolated from metastatic breast cancer MDA-MB-231 cells compared to a non-metastatic cell line, which increased tumor progression by targeting Cyclin-G2. It also increased resistance to docetaxel, epirubicin, or gemcitabine [129]. Exosomes isolated from MDA-MB-231 and BT549 cells had higher expression of miR-425-5p when compared to human mammary fibroblasts (HMFs), which increased cell proliferation and migration by increasing the expression of *α*-SMA FAP*α*, CXCL1, TGFB1, and IL-6. In addition, the expression of vimentin, N-cadherin, fibronectin, and MMP2 was also increased. This suggests that miR-425-5p mediates the interaction between breast cancer cells and stroma [130]. The downregulation of miR-7-5p in breast cancer cells promoted the expression of the atypical WNT pathway by increasing the phosphorylation of JNK and c-Jun proteins, which in turn decreased the expression of ZEB1, facilitating the expression of EMT genes. miR-7-5p binds to the 3’-UTR region of RYK gene to increase the invasive capacity of breast cancer cells [131]. When internalized by MCF-7 cells, human umbilical cord mesenchymal-stem-cell-derived exosomes inhibited migration and invasion by downregulating the expression of ZNF367 and upregulating miR-21-5p expression. A miR-21-5p mimic partially abolished ZNF367-induced migration and invasion [132]. MCF10A cocultured with EVs isolated from the HCC1806 TNBC cell line showed increased cell proliferation and resistance to therapeutic agents. Gene and miRNA expression profiling revealed that 138 genes and 70 miRNAs that were differentially expressed were involved in PI3K/AKT, MAPK, and HIF1A pathways. The KEGG pathway analysis of these 10 genes showed their involvement in cancer and signaling pathways regulating the pluripotency of stem cells [133]. The impact of macrophage-derived exosomal miR-503-3p on the development of breast cancer was studied by Huang et al. (2021). Treatment with exosomes upregulated the expression of β-catenin, Glut1, LDH-A, and DACT2, while p-β-catenin was downregulated. The ratio of cells in the S phase increased, while cells in the G0/G1 phase decreased, leading to increased cell proliferation. In xenograft models, the tumor weight and volume were increased in the exosome-treated group when compared to controls [134].

#### 1.4.2. Role of Exosomal miRNAs in Cancer-Associated Fibroblasts in Breast Cancer

Triple-negative breast cancer (TNBC)-derived exosomes and their miRNA cargoes may affect the conversion of fibroblasts into a cancer-associated fibroblast (CAF)-like phenotype in the tumor microenvironment. Exosomal miR-185-5p, miR-652-5p, and miR-1246 synergistically activated stromal fibroblasts to a pro-migratory functional state by upregulating the expression of FAP, MCT4, and Caveolin-1. It also promoted fibroblast collagen contraction and increased the expression of FAK, which contributed to increased invasion and metastasis [135]. Among the 31 differentially expressed miRNAs, miR-500a-5p was highly expressed in both MDA-MB-231 and MCF7 cells when incubated with CAF-derived exosomes, which contributed to overall survival in patients. The expression of EMT-related markers increased, while the expression of N-cad, FN1, ZEB1, snail, and slug was reduced by binding to ubiquitin-specific peptidase 28. In vivo, miR-500a-5p increased breast cancer cell proliferation, and Western blotting showed an inverse relationship between miR-500a-5p, USP28, and E-cad. High levels of N-cadherin, fibronectin, slug, and vimentin increased lung metastasis [136]. miR-18b was upregulated in CAFs isolated from MDA-MB-231 breast cancer cells. This induced cell migration and metastasis by binding to the 3’-UTR of Transcription Elongation Factor A-Like 7 (TCEAL7) and decreasing its expression. Increased expression of NF-κB, MMP9, and ICAM-1 and snail contributed to metastasis. In a xenograft model, miR-18b-loaded exosomes had a cancer-promoting effect by increasing the expression of MMP2 and MMP9. In these mice, metastatic nodules in the lung were significantly higher than in the control group [137]. Exosomes isolated from the supernatant of monocytes educated by the culture media derived from CAFs or normal fibroblasts (NFs) showed that the upregulation of miR-181a promoted tumorigenicity in vivo by modulating the PTEN/AKT signaling axis [138].

#### 1.4.3. Association of Exosomal miRNAs with Hormone Receptor Status in Breast Cancer

Exosomes are associated with receptor status in breast cancer. miR-101, miR-372, and miR-373 in exosomes were higher in breast cancer patients than in normal samples, which was associated with overall survival. Dysregulated expression of miR-93, miR-155, and miR-373 was linked to metastasis, proliferation, cell migration, and apoptosis. The levels of miR-20a and miR-30b were higher in primary than in recurrent breast cancer patients. miR-16, miR-30b, and miR-93 were increased in DCIS patients compared with healthy women. miR-16 was also positively associated with estrogen and progesterone receptor status. Triple-negative patients had lower levels of miR-16 than receptor-positive patients [139]. Luminal carcinoma patients had lower levels of miR-373 than triple-negative patients. miR-373 was also associated with hormone receptor status. Overexpression in MCF-7 cells decreased the expression of estrogen receptors and inhibited apoptosis [140]. hsa-miR-576-3p was significantly upregulated in exosomes isolated from breast cancer patients with metastasis, while hsa-miR-130a-3p was downregulated. In HER2-positive samples, hsa-miR-197-3p, -410-3p, and -490-3p were increased, while hsa-miR-32-5p was only increased in TNBC, and Hsa-miR-125a-3p was increased in the ER/PR+ group. Exosomal hsa-miR-342-3p was inversely correlated with grading and hsa-miR-340-5p levels [141].

#### 1.4.4. Role of Exosomal miRNAs in Chemo- and Radioresistance in Breast Cancer

miRNAs in exosomes enable cells to communicate with each other and respond to therapies. To identify the miRNAs involved in chemotherapy, exosomes were isolated from patients before and after chemotherapy with doxorubicin and paclitaxel. Chemotherapy increased the levels of exosomal miR-378a-3p and miR-378d. In vitro studies showed that these miRNAs were transferred to neighboring cells, where they activated WNT and NOTCH pathways and induced drug resistance by targeting Dickkopf 3 (DKK3) and NUMB. Overexpression of miR-378a-3p or miR-378d decreased both proteins, whereas the expression of stemness-associated proteins was increased. The coculture of chemotherapy-elicited exosomes with cancer cells decreased the levels of NUMB and DKK3, increased the expression of CD44 and ALDH1A1, and activated WNT/β-catenin and Notch signaling pathways [142]. Exosomal miRNAs from trastuzumab-resistant and -sensitive patients revealed that miR-1246 and miR-155 were upregulated. miR-1246 had 78.1% sensitivity and 75% specificity, while miR-155 had 68.8% sensitivity and 97.2% specificity in discriminating resistant from sensitive patients. High levels of both miRNAs predicted poor event-free survival for early-stage and metastatic patients [143]. The exposure of breast cancer cells to chemotherapeutic agents induced the production of miR-9-5p, miR-195-5p, and miR-203a-3p, which enabled cells to survive in a chemotoxic environment. These miRNAs decreased the expression of the transcription factor One Cut Homeobox 2 (ONECUT2), leading to the increased expression of genes associated with stemness, such as NOTCH1, SOX9, NANOG, OCT4, and SOX2. The increased expression of ONECUT2 or the decreased expression of these miRNAs was able to reverse these effects [144]. Exosomal miR-567 levels were significantly decreased in trastuzumab-resistant patients when compared with sensitive ones. Overexpression reversed chemoresistance, and when packaged into exosomes and transferred to neighboring cells, it induced trastuzumab resistance and suppressed autophagy by targeting ATG5 [145]. The exosomal microRNA profile of a cisplatin-resistant TNBC cell line (MDA-MB-231) showed the differential expression of miR-221-3p, miR-196a-5p, miR-17-5p, and miR-126-3p, targeting genes such as ATXN1, LATS1, GSK3β, ITGA6, JAG1, and MYC, which increased the CSC-like cell subpopulation, contributing to cisplatin resistance [146]. Circulating tumor cells and serum exosomal miRNAs isolated from blood samples from women with localized breast cancer undergoing neoadjuvant chemotherapy showed higher expression of miR-21 and miR-105 in breast cancer samples than in controls, and the levels were different between patients with localized breast cancer and metastatic breast cancer. Before neoadjuvant therapy, exosomal miRNA-21 and miRNA-105 expression levels were higher in larger tumors than in small tumors and were associated with hormone receptor status [147]. The analysis of miRNAs from exosomes isolated from the serum of stage I–III breast cancer in the same patients before and after chemotherapy revealed the elevation of miR-3662 in well-differentiated and moderately differentiated breast carcinoma, while miR-146a was high in patients with tumor sizes >2 cm, which significantly decreased after chemotherapy. miR-3662, miR-146a, and miR-1290 expression levels were high in patients with lymph node metastasis, and patients with stage I had lower levels of these miRNAs than patients with stages II/III [148]. Exosomes isolated from tamoxifen-resistant MCF-7/TAMR-1 (M/T) cells showed that miRNA-205 had the greatest increase among the miRNAs analyzed (miR-181a-5p, miR-21-3p, miR-125b, miR-200c, miR-205, and miR-99a). Coculturing M/T-Exo with breast cancer cells increased cell proliferation, invasion, and migration and suppressed apoptosis by activating the caspase pathway, phosphorylating AKT, and targeting transcription factor 1 (E2F1). Mice that received an intratumoral injection of M/T-Exo showed increased tumor size, resistance to tamoxifen, and changes in E2F1 protein [149].

#### 1.4.5. Anticancer Effects of Exosomal miRNAs on Breast Cancer

Exosomes carrying miR-16-5p isolated from the bone marrow of patients impaired cell proliferation invasion, and migration, enhanced apoptosis, and decreased EMT in breast cancer cells by downregulating the expression of EPHA1 and NF-κB signaling. Nude mice xenografted with tumors had a smaller tumor volume and lower weight in the miR-16-5p group when compared to the control [150]. Exosomal miR-551b-3p could be transferred to MDA-MB-231 cells from BMSCs, which suppressed cell proliferation and migration and induced apoptosis by downregulating the expression of tripartite motif-containing protein 31 (TRIM31). Xenograft mice showed decreased tumor growth and the downregulation of TRIM31 and AKT, indicating that exosomal miR-551b-3p could be a tumor suppressor [151]. The differential expression of miR-7-5p was found in breast cancer cell lines with different invasive properties. It reduced migration and invasion by targeting RYK and decreasing the phosphorylation of JNK. The reduced phosphorylation of JNK led to the decreased phosphorylation of the c-Jun protein, which in turn inhibited the EMT process to suppress the invasion of breast cancer [131]. The expression of miR-940 was found to be significantly downregulated in exosomes isolated from the serum of breast cancer patients. It was lower in HER2/neu-positive patients than in HER2/neu-negative patients and higher in patients without lymph node metastasis than in those with metastasis, suggesting that the expression of miR-940 is related to metastasis and HER2/neu status [152]. miR-134-5p was expressed at low levels in breast cancer cells, and overexpression of exosomal miR-134-5p suppressed the proliferation, migration, and invasion of breast cancer cells by inducing cell cycle arrest in the G0/G1 phase and inhibiting the cell number in the S phase. miR-134-5p acted as a tumor suppressor by targeting ARHGAP1 and inhibiting the activity of the PI3K/AKT pathway [153]. miR-3182-loaded exosomes significantly inhibited the viability of MDA-MB-231 cells, increased their doubling time, and decreased colony formation. It increased the number of cells in the G0/G1 phase, decreased the number of cells in the S and G2/M phase, and increased the number of apoptotic cells when compared to the control by targeting the mTOR pathway [154].

### 1.5. Prostate Cancer

Prostate cancer is the fifth leading cause of cancer death among men, with an estimated 1.4 million new cases and 375,000 deaths worldwide. In the early stages, it is asymptomatic, while patients in the later stages have symptoms of urination and hematuria, among others [30]. The measurement of serum prostate-specific antigen (PSA) is the current method of diagnosis but is not very reliable due to its low sensitivity and poor prediction [155]. Therefore, new methods of diagnosis are needed that would provide an accurate prognosis and diagnosis. The elevated/decreased levels of certain miRNAs in exosomes can be used as sensitive and accurate surrogate markers not only to diagnose the disease but also to potentially provide information on prostate cancer initiation, progression, and the morbidities associated with cancer treatment.

#### 1.5.1. Role of Exosomal miRNAs in Cell Signaling in Prostate Cancer

Dysregulated miRNAs are involved in almost all signaling pathways associated with cancer progression. The profiling of exosomes from the serum of aggressive prostate cancer, benign prostatic hyperplasia (BPH), and disease-free controls was performed to identify a biomarker that could have diagnostic potential. miR-1283, miR-1246, miR-26b-5p, and miR-302c-3p were significantly upregulated, and 16 miRNAs were downregulated in aggressive prostate cancer when compared with BPH. Three miRNAs, miR-1246, miR-766-3p, and miR-302c-3p, were dysregulated in aggressive prostate cancer when compared to benign and normal samples. miR-1246 expression was correlated with the pathologic grade, metastasis, and poor prognosis. The expression increased by 31-fold in aggressive prostate cancer and 23-fold in BPH when compared to controls, which can be used to distinguish the different stages of the disease [156]. In prostate cancer patients, exosomal miR-1290, -1246, and -375 were associated with the overall survival rate. Patients with high levels of miR-1290 and -375 had a mortality rate of 80% in the 20-month follow-up period when compared to controls [157]. The comparison of miRNAs between EVs and whole plasma between prostate cancer patients and BPH revealed that miR-375 was significantly increased in PC patients when compared to BPH. miR-200c-3p and miR-21-5p were differentially expressed between PC and BPH samples. The level of Let-7a-5p was significantly decreased in EVs from PC patients with high Gleason scores when compared to patients with low Gleason scores [158]. miR-141 was found to be upregulated in prostate cancer and could be used as a biomarker. The expression was higher in serum exosomes from prostate cancer patients than in those from benign prostate hyperplasia (BPH) patients and controls. The expression was also higher in metastatic samples than in localized prostate cancer samples, which could be used to differentiate between the two [159]. Shin et al. found that five miRNAs (miR-451, miR-142-3p, miR-16, miR-21, and miR-140-3p) were upregulated and one (miR-636) was downregulated in the metastatic group when compared to localized prostate tumor patients. Prostate Cancer Metastasis Risk Scoring was used to assess the survival rates and showed that patients with high scores had poorer recurrence-free survival than those with low scores [160]. High-throughput sequencing of exosomes collected from the plasma of PCa patients identified 94 differentially expressed miRNAs when compared to healthy controls. These miRNAs were involved in cell migration, cell adhesion, TGFβ signaling, metabolic pathways, and ECM organization. qPCR results confirmed the upregulation of miR-127-5p and miR-217 and the downregulation of miR-148a-3p and miR-23b-3p. In vitro and in vivo studies revealed that exosomal miR-217 promoted cell proliferation and invasion, while miR-23b-3p inhibited cell proliferation and invasion. Western blots showed the altered expression of vimentin and E-cadherin, confirming the role of these miRNAs in EMT [161]. Exosomes containing miR-95, when taken up by prostate cancer cell lines, promoted tumor formation by binding to JunB. In PCa samples, JunB expression was inversely correlated with miR-95, and higher miR-95 expression resulted in worse clinicopathological features. TAM-mediated PCa progression is partially attributed to the aberrant expression of miR-95, and the miR-95/JunB axis could be used for cancer therapy, which confirms that tumor-associated macrophages take part in cell-to-cell communication [162]. Increased expression of hsa-mir-200b and hsa-mir-331-3p was found in exosomes isolated from the saliva of patients with prostate cancer. Both miRNAs showed a positive predictive value of 71% in prostate cancer patients. hsa-mir-200b increased cell proliferation and migration by activating Bmi-1, while hsa-mir-331-3p acts by targeting the ERBB-2 signaling pathway. In 70% of the cases, increased miRNAs in salivary exosomes correlated with histologic findings in tissues, making exosomal miRNAs a reliable diagnostic marker [163]. Exosomes isolated from prostatic fluids from patients with prostate cancer had significantly higher expression of miR-20b-5p when compared to control samples and reduced expression of retinoblastoma-associated protein 1 (RB1), which is a tumor suppressor [164]. RT-qPCR results confirmed the differential expression of miR-205-5p, miR-148a-3p, miR-125b-5p, miR-183-5p, and miR-425-5p in exosomes of PCa. miR-425-5p was associated with residual tumor, N and T tumor stages, and p53 status, while miR-183-5p was positively associated only with the T stage. miR-425-5p acted by targeting the small heat-shock protein HSPB8 [165].

The expression of miR-181a-2, miR-572, miR-107, and miR-574-3p can be used to distinguish prostate cancer patients without metastasis from control samples. The expression levels of miR-181a-2 and miR-572 were significantly decreased in the exosomes of prostate cancer patients, while miR-107 and miR-574-3p were increased [166]. In patients with PCa and BPH, plasma miR375 was able to distinguish between the two, while miR-200c-3p and miR-21-5p were able to distinguish EVs. The let-7a-5p level was able to distinguish between patients with Gleason scores ≥ 8 vs. ≤6. It decreased in patients with high Gleason scores (≥8) compared to low Gleason scores (≤6) [158]. The differential expression of miR-23b-3p, miR-27a-3p, miR-27b-3p, miR-1-3p, miR-10a-5p, and miR-423-3p was found in EVs isolated from patients with grade 4 or higher and grade 3 or lower. Grade 4 or higher patients had increased expression of miR-23b-3p, miR-27a-3p, and miR-27b-3p, while the expression of miR-1-3p, miR-10a-5p, and miR-423-3p was decreased, which could be used to differentiate different grades of cancer [167]. miR-let-7c was found to be downregulated in metastatic PCa and high-grade-group patients when compared to controls, and when packaged into exosomes, it significantly decreased the proliferation and migration of PC3 and CWR22Rv1 cells [168]. The decreased expression of miR-205 in the serum exosomes of prostate carcinoma patients was negatively associated with the cancer stage, metastasis, and the PSA level at the time of initial diagnosis. Overexpression of miR-205 reversed the above-mentioned functions [169]. miR-423-5p expression in the serum exosomes of PCa patients was found to be higher than in control samples, indicating that it may play a role in cancer progression. Microarray and luciferase reporter assay results confirmed that it targeted FRMD3 and downregulated its expression. Overexpressing miR-423-5p increased cell proliferation and migration in vitro, and when inhibited, it decreased EMT, as evidenced by the decreased expression of vimentin and N-cadherin and the increased expression of E-cadherin. The same pattern was observed in xenografts injected with exosomal miR-423-5p, suggesting that it could serve as a reference for PCa therapy [170]. miRNAs have been associated with biochemical recurrence (BCR) and metastasis in prostate cancer. miR-26a-5p, miR-532-5p, and miR-99b-3p were upregulated in exosomes from BCR patients when compared to controls, and these patients had lower survival rates than patients expressing low levels of these miRNAs. Multivariate analysis showed that miR-532-5p expression was the only significant factor for prostate cancer recurrence [171]. High levels of circulating levels of miR-424-positive EVs were found in patients with metastatic prostate cancer than in patients with primary tumors and BPH. EVs containing miR-424 promoted stem-cell-like properties and enhanced tumorigenesis. Similar results were obtained in a xenograft mouse model [172].

Hypoxia is associated with disease progression and treatment failure in prostate cancer. The role of miRNAs in a hypoxic environment was studied by Panigrahi et al., who found increased expression of miR-885 and decreased expression of miR-521 in a cell line and the serum exosomes of PCa patients. These patients showed inverse correlations with the tumor T stage, N stage, and Gleason score. This differential expression could be potentially used as a biomarker [173]. The effects of exosomal miR-183 on cell proliferation, invasion, and migration were evaluated by Dai et al. using LNCaP cells. High expression of miR-183 was found in prostate cancer, and its carcinogenic effects were mediated by the downregulation of the expression of TPM1 [174]. Exosomes can also be used to differentiate between bulk and cancer stem cells in prostate cancer. Exosomes isolated from bulk and cancer stem cell populations showed 19 differentially expressed miRNAs, out of which 6 were overexpressed in CSCs and 13 were overexpressed in bulk exosomes. These were involved in cell proliferation, invasion, and migration and osteoblast differentiation, which collectively collaborate in prostate carcinogenesis and could be used as potential biomarkers and therapeutic targets [175]. Exosomes isolated from DU145 and PC-3 cells had higher expression of miR-21 and miR-574-3p when compared to RWPE-1 cells, which could be used as diagnostic biomarkers in prostate cancer. The higher expression of miR-21 and miR-574-3p seen in PC-3 exosomes correlated with a higher metastatic potential of PC-3 cells. Western blots of EpCAM, EGFR, survivin, and IGF-1R, showed higher expression in exosomes from PCa than in controls [176]. Overexpression of miR-153 in high-GS cell lines-controlled cell proliferation, migration, and invasion by targeting Kruppel-like factor 5 (KLF5). A target scan revealed three conserved sites where KLF5 could bind and exert its carcinogenic effect. Secreted miR-153 in exosomes was able to influence cellular growth as well. In patients, high expression of miR-153 was associated with a poor prognosis [177].

#### 1.5.2. Role of Urinary Exosomal miRNAs in Prostate Cancer

Urinary exosomes can be used as biomarkers, which could enable a convenient and noninvasive approach to diagnosing PCa. Since urine composition reflects changes in the urogenital system, it is an invaluable source for investigating neoplastic abnormalities in PCa. Urinary exosomal miRNAs can be used as noninvasive markers for predicting metastasis and prognosis in PCa patients. The roles of five miRNAs, miR-21, miR-141, miR-214, miR-375, and let-7c, that are frequently deregulated in prostate cancer were studied in urinary exosomes by Fogi et al., who found the upregulation of miR-21, miR-375, and let-7c in patients with prostate cancer. The expression of miR-21, miR-141, and miR-214 was used to distinguish between healthy subjects and low-risk groups versus intermediate- and high-risk groups [178]. Hydrogel-based hybridization chain reaction (HCR) for multiplex signal amplification from urinary exosomes revealed the downregulation of miR-3665 and the upregulation of miR-6090 in prostate cancer patients, which could be used as biomarkers [179]. miR-19b in urinary exosomes was able to identify PCa patients from normal samples with 100% specificity and 93% sensitivity, which could be used as a primary or auxiliary criterion for the diagnosis of prostate cancer [180]. The differential expression of hsa-miR-532-3p and hsa-miR-6749-5p was found in urinary exosomes isolated from PCa compared to BPH, which could be used to differentiate the two. miR-532-3p was upregulated by 1.6-fold, and hsa-miR-6749-5p had a 2-fold increase in the PCa group when compared to BPH patients [181]. miR-30b-3p and miR-126-3p, which were overexpressed in the urinary EVs of PCa patients, were better predictors than serum PSA [160]. Four miRNAs, miR-572, miR-1290, miR-141-5p, and miR-145-5p, which are markers of PCa, were measured in the urinary EVs of cancer patients, BPH patients, and controls. Although significant differences were found between controls and patients, only miR-145 could be used to distinguish between PCa and BPH patients. Patients with Gleason scores ≥8 had significantly higher expression than patients with Gleason scores ≤7. The receiver-operating characteristic curve (ROC) revealed that miR-145 in UEVs combined with serum PSA could differentiate PCa from BPH better than PSA alone [182]. miR-30b-3p and miR-126-3p were overexpressed in the urinary EVs of PCa patients compared with biopsy-negative men, which could be associated with the prediction of PCa in biopsy specimens. The sensitivity of these miRNAs in EVs was higher than that in serum, making EVs a better candidate for the prediction of PCa [183]. Next-generation sequencing (NGS) results of urinary exosomes from prostate cancer patients validated by qPCR revealed higher expression of miR-451a, miR-486-3p, and miR-486-5p, in contrast to miR-375, whose expression was decreased when compared to control samples. miR-375 showed a correlation with the clinical T stage and bone metastasis and could differentiate between localized and metastatic PCa [184]. miR-2909 was found to be present in the urinary exosomes of prostate cancer patients irrespective of the sensitivity to hormones. It showed a significant correlation with the Gleason score, indicative of the severity of the disease, and could potentially be used as a noninvasive biomarker for prostate cancer [185].

#### 1.5.3. Role of Exosomal miRNAs in Osteoblastic Modulation in Prostate Cancer

miR-141-3p is involved in the microenvironment of bone metastases in PCa cancer. The level of miR-141-3p in exosomes in a PCa cell line was higher than in the control, and a confocal microscope showed that they were able to enter osteoblasts, which led to increased osteoblast activity and osteoprotegerin (OPG) expression and decreased the expression of DLC1, indicating the functional significance of this miRNA in activating the p38MAPK pathway. In vivo experiments showed that exosomal miR-141-3p had bone-targeting specificity, promoted osteoblast activity, and increased bone metastasis [186]. The role of miR-148a in bone metastasis in PCa was studied by Tian et al. (2021), where PC3 exosomes were used. When osteoclasts were cultured with PC3 exosomes, osteoclast differentiation was affected, and the genes associated with osteoclastic maturation were downregulated. In addition, the expression of miR-148a was downregulated, and mTOR and AKT signaling pathways were blocked, indicating the relationship between miRNA and signaling pathways [187]. Exosomal transfer of miR-1275 from PCa cells to osteoblasts significantly reduced the proliferation of osteoblasts and the expression levels of osteoblast-specific genes. It increased the expression of RUNX2, which is the regulator of osteoblast activity, by downregulating SIRT2. This study confirms that exosomal miR1275 promotes the proliferation and activity of osteoblasts via modulation of SIRT2/Runx2 signaling [188]. Exosomal miRNAs such as miR-100-5p and miR-21-5p from PCa can affect the cancer-related microenvironment and metastatic niche. Increased expression of the above two miRNAs increased the expression of MMP-2, -9, and -13. In addition, the expression of RANKL and fibroblast migration increased, leading to increased cell migration [175]. miR-141 levels in exosomes isolated from PCa patients can be used to distinguish between patients with metastatic and localized cancer. The expression of miR-141 was higher in metastatic cancer than in BPH and controls. The receiver-operating characteristic curve revealed that the serum exosomal miRNA was 87% sensitive in differentiating between the two [159]. hsa-miR-940 in exosomes from osteoblastic-phenotype-inducing prostate cancer cell lines promoted the osteogenic differentiation of human mesenchymal stem cells by targeting ARHGAP1 and FAM134A and downregulating their expression. When exosomes from the miR-940–overexpressing MDA-MB-231 cells were implanted on the calvarial bones of immunodeficient mice, the tumors had extensive mineralized tissues composed of bone matrix and surrounded by osteoblast-like cells or osteoids. When injected into the tibiae of mice, they induced osteoblastic lesions, proving that the involvement of exosomal miRNA secreted by cancer cells in the bone microenvironment can induce osteoblastic bone metastasis [189]. Exosomes from the prostate cancer cell line PC-3 dramatically inhibited osteoclast differentiation by downregulating miR-214 and osteoclast genes such as CTSK, NFATc1, and ACP5 and blocked the NF-κB signaling pathway. Elevating miR-214 levels in the bone metastatic site may attenuate the invasion of prostate cancer [190].

#### 1.5.4. Role of Exosomal miRNAs in Castration-/Chemoresistance in Prostate Cancer

Exosomal has-miR-148a-3p can be used as a potential prognostic marker in castration-resistant prostate cancer (CRPC) patients. According to the PSA level, patients were divided into different groups, namely, P0, P1, P2, and P3, indicating the start of therapy, the response to therapy, the initial increase in PSA levels, and elevated PSA levels, respectively. Significant differences in hsa-148a-3p levels were found between periods P1 and P3 and between P0 and P3 [191]. Neuroendocrine prostate cancer (NEPC) is a highly aggressive variant of castration-resistant prostate cancer (CRPC) that is mostly seen after treatment with androgen pathway inhibitors. To identify a noninvasive EV-based biomarker, next-generation sequencing was carried out on the serum of samples from CRPC patients with adenocarcinoma characteristics (CRPC-Adeno) vs. CRPC-NE. The results identified the significant dysregulation of 182 known and 4 novel miRNAs. miR-891a-5p, -9-3p, -877-5p, -592, -200a-3p, and 1180-3p were found to be upregulated, while miR-152-3p, -28-5p, -378d, and -23a-3p were found to be decreased in CRPC-NE tissues. Western blotting showed that NEPC samples had higher levels of thrombospondin 1 (TSP1), which could be used as a biomarker to identify NEPC [192]. Docetaxel resistance limits the successful treatment of castration-resistant prostate cancer, in which exosomes may play a role. Of the several miRNAs that were either up- or downregulated, only miR-34a had clinical significance. Further experiments proved that it regulates the expression of BCL-2, which may take part in docetaxel resistance [193]. Cancer-associated fibroblasts (CAFs) promote the metastasis of PCa after androgen deprivation therapy through the transfer of exosomal miR-146a-5p to PCa cells. miR-146a-5p levels in exosomes were significantly decreased after the cells were treated with ethanol to mimic the castration microenvironment after ADT. The loss of exosomal miR-146a-5p promoted EMT by activating the EGFR/ERK pathway [194]. miR-375 expression was found to be increased in PCa tissues and exosomes, which correlated with the Gleason score. Higher expression increased EMT markers and enhanced cell migration proliferation and enzalutamide resistance. Silencing the expression of miR-375 upregulated PTPN4 and downregulated p-STAT3, leading to decreased cell proliferation. The upstream target PTPN4 could be used as an alternative therapeutic strategy for PCa [195].

To identify how miRNAs are involved in resistance in prostate cancer, exosomes were isolated from the metastatic PCa cell lines DU145 and PC3 and their PTX-resistant versions DU145-TXR and PC3-TXR. Microarray analysis showed that hsa-miR-141-3p, hsa-miR-429, hsa-miR-192-5p, hsa-miR-192-3p, hsa-miR-606, hsa-miR-3176, hsa-miR-1224-3p, hsa-miR-381-3p, hsa-miR-933, and hsa-miR-34b-3p were downregulated, and 19 miRNAs were upregulated. These were involved in ErBb, mTOR, WNT, and actin cytoskeleton pathways, among others. AR and PTEN pathways upregulated the miRNAs hsa-miR-16-5p, -23c, -32-5p, -3915, -5004-5p, -488-3p, -3673, and -3654 and downregulated the miRNAs hsa-miR-3176 and -141-3p. TCF4 target genes upregulated hsa-miR-32-5p and downregulated the miRNAs hsa-miR-141-3p, -606, -381, and -429. Depending on the pathways involved, detailed mechanisms can be studied that could be responsible for chemoresistance [196]. The role of miR-1182 in the docetaxel (DTX) resistance of PCa was studied by Zhang et al. (2021). They found that miR-1182 was downregulated in DTX-resistant PCa tissue specimens and cell lines. TPD52 was the target of miR-1182, and its upregulation weakened the promotive effect of miR-1182 on DTX sensitivity. Increased expression of Circ-XIAP directly targeted miR-1182, and its inhibition rendered the cells sensitive to DTX. This study revealed that the miR-1182/TPD52 axis may provide a promising therapeutic target for PCa chemotherapy [197]. miR-27a participated in chemoresistance by restraining the expression of the P53 gene. In silico analysis showed that the 3′-UTR of p53 mRNA possesses two putative binding sites for miR-27a. miR-27a binding to P53 downregulated its expression and rendered cells sensitive to cisplatin, DOX, and docetaxel [198]. The Met/miR-130b axis in exosomes can be used as a noninvasive tool for PCa surveillance and therapy monitoring. Increased c-Met activation increased the expression of miR-130b, inhibited androgen receptor expression, promoted metastasis, and increased resistance to hormone ablation therapy. In patients, high expression correlated with a high Gleason score and pT and tumor recurrence after 10 yrs [199].

#### 1.5.5. Exosomal miRNAs as Tumor Suppressor in Prostate Cancer

Exosomal miR-26a derived from prostate carcinoma cells had a suppressive effect on the metastasis and tumor growth of prostate carcinoma. It significantly altered the expression of EMT genes to counteract the invasion and migration of cells caused by PC-3 exosomes. miR-26a mimics downregulated the expression of MMP-2, MMP-9, and TIMP-2 to prevent the invasion of cells. Xenografts in mice showed that miR-26a mimics could increase the expression of miR-26a, ultimately leading to decreased tumor size and metastasis [200]. Exosomes isolated from prostate-cancer-associated fibroblasts carrying miR-423-5p increased the resistance of prostate cancer cells to taxane treatment. It inhibited the expression of GREM2 through the TGF-β pathway. TGF-β inhibition was able to partially reverse cell proliferation and colony formation. The inhibition of miR-423-5p in vivo prevented tumor growth, tumor weight, and taxane resistance and increased GREM2 expression [201]. Increased exosomal circHIPK3 expression in the serum of PCa increased the severity of the disease by decreasing the expression of miR-212, which acts as a tumor suppressor. miR-212 exerted this tumor suppressor effect by regulating the expression of BMI-1 expression. The regulation of the miR-212/BMI-1 axis can be exploited in cancer therapy [202]. miR-187 expression was found to be elevated in hBMSCs and hBMSC-exos treated with a miR-187 mimic. When hBMSC-exos were cocultured with PC3 cells, the proliferative, invasive, and migratory capacities of PC-3 cells were significantly reduced. Western blotting showed the elevated expression of E-cadherin and Bax, while CD276, Bcl-2, and JAK3-STAT3-Slug-pathway-related genes showed decreased expression, leading to repressed tumor growth in mice. hBMSC-exos overexpressing miR-187 may function as a promising target for prostate cancer treatment [203].

## 2. Conclusions and Future Perspectives

Research over the years suggests that exosomes are involved in normal homeostasis and are deregulated in diseases such as cancer. They can act as tumor suppressors or enhancers and are involved in mediating intracellular communication within the tumor cell and between tumor cells and the environment. The field of exosomes, although relatively new, is progressing at rapid speed with new discoveries and a broad range of applications in the use of diagnostic and prognostic markers. Exosomes are also being tested as vehicles for drug delivery for shRNAs targeting oncogenic proteins. Although progress has been made in exosome research, not much is known about how to realize exosome biogenesis, control exosome release, and address their heterogeneity and stability for longer shelf life. In addition, there are no uniform extraction methods or routes of administration, and very little is known about the immune response. As of now, there are no standardized methods for clinical settings that would allow the research to be taken from bench to bedside since most of the research is conducted in vitro. The clinical application of exosomes is an emerging field of study, and further investigations must consider the cost and time for the large-scale production of exosomes, their tailored preparation as vehicles for drug delivery, and their cytotoxicity. All these evaluations should be made to protect patient safety and avoid undesirable side effects before market approval. Moreover, understanding the physiological and pathological effects is crucial in developing new therapeutic strategies.

## References

[1] Sheta M., Taha E.A., Lu Y., Eguchi T. (2023). Extracellular Vesicles: New Classification and Tumor Immunosuppression. Biology.

[2] Willms E., Cabañas C., Mäger I., Wood M.J.A., Vader P. (2018). Extracellular Vesicle Heterogeneity: Subpopulations, Isolation Techniques, and Diverse Functions in Cancer Progression. Front. Immunol..

[3] Wei H., Chen Q., Lin L., Sha C., Li T., Liu Y., Yin X., Xu Y., Chen L., Gao W. (2021). Regulation of exosome production and cargo sorting. Int. J. Biol. Sci..

[4] Elliott R.O., He M. (2021). Unlocking the Power of Exosomes for Crossing Biological Barriers in Drug Delivery. Pharmaceutics.

[5] Gurung S., Perocheau D., Touramanidou L., Baruteau J. (2021). The exosome journey: From biogenesis to uptake and intracellular signalling. Cell Commun. Signal..

[6] Nicolini A., Ferrari P., Biava P.M. (2021). Exosomes and Cell Communication: From Tumour-Derived Exosomes and Their Role in Tumour Progression to the Use of Exosomal Cargo for Cancer Treatment. Cancers.

[7] Osaki M., Okada F. (2019). Exosomes and Their Role in Cancer Progression. Yonago Acta Med..

[8] Larios J., Mercier V., Roux A., Gruenberg J. (2020). ALIX- and ESCRT-III-dependent sorting of tetraspanins to exosomes. J. Cell. Biol..

[9] van Niel G., Carter D.R.F., Clayton A., Lambert D.W., Raposo G., Vader P. (2022). Challenges and directions in studying cell-cell communication by extracellular vesicles. Nat. Rev. Mol. Cell Biol..

[10] Hsu M.T., Wang Y.K., Tseng Y.J. (2022). Exosomal Proteins and Lipids as Potential Biomarkers for Lung Cancer Diagnosis, Prognosis, and Treatment. Cancers.

[11] Ferreira J.V., da Rosa Soares A., Ramalho J., Máximo Carvalho C., Cardoso M.H., Pintado P., Carvalho A.S., Beck H.C., Matthiesen R., Zuzarte M. (2022). LAMP2A regulates the loading of proteins into exosomes. Sci. Adv..

[12] Zhang J., Li S., Li L., Li M., Guo C., Yao J., Mi S. (2015). Exosome and exosomal microRNA: Trafficking, sorting, and function. Genom. Proteom. Bioinform..

[13] Li C., Zhou T., Chen J., Li R., Chen H., Luo S., Chen D., Cai C., Li W. (2022). The role of Exosomal miRNAs in cancer. J. Transl. Med..

[14] Ala U. (2020). Competing Endogenous RNAs, Non-Coding RNAs and Diseases: An Intertwined Story. Cells.

[15] Zietzer A., Hosen M.R., Wang H., Goody P.R., Sylvester M., Latz E., Nickenig G., Werner N., Jansen F. (2020). The RNA-binding protein hnRNPU regulates the sorting of microRNA-30c-5p into large extracellular vesicles. J. Extracell. Vesicles.

[16] Jeppesen D.K., Fenix A.M., Franklin J.L., Higginbotham J.N., Zhang Q., Zimmerman L.J., Liebler D.C., Ping J., Liu Q., Evans R. (2019). Reassessment of Exosome Composition. Cell.

[17] Lu P., Li H., Li N., Singh R.N., Bishop C.E., Chen X., Lu B. (2017). MEX3C interacts with adaptor-related protein complex 2 and involves in miR-451a exosomal sorting. PLoS ONE.

[18] Li X., Corbett A.L., Taatizadeh E., Tasnim N., Little J.P., Garnis C., Daugaard M., Guns E., Hoorfar M., Li I.T.S. (2019). Challenges and opportunities in exosome research-Perspectives from biology, engineering, and cancer therapy. APL Bioeng..

[19] O’Brien K., Breyne K., Ughetto S., Laurent L.C., Breakefield X.O. (2020). RNA delivery by extracellular vesicles in mammalian cells and its applications. Nat. Rev. Mol. Cell Biol..

[20] Zhang Y., Liang F., Zhang D., Qi S., Liu Y. (2023). Metabolites as extracellular vesicle cargo in health, cancer, pleural effusion, and cardiovascular diseases: An emerging field of study to diagnostic and therapeutic purposes. Biomed. Pharm..

[21] Gurunathan S., Kang M.H., Jeyaraj M., Qasim M., Kim J.H. (2019). Review of the Isolation, Characterization, Biological Function, and Multifarious Therapeutic Approaches of Exosomes. Cells.

[22] Vidal M. (2019). Exosomes: Revisiting their role as “garbage bags”. Traffic.

[23] Varela-Eirin M., Varela-Vazquez A., Rodríguez-Candela Mateos M., Vila-Sanjurjo A., Fonseca E., Mascareñas J.L., Eugenio Vázquez M., Mayan M.D. (2017). Recruitment of RNA molecules by connexin RNA-binding motifs: Implication in RNA and DNA transport through microvesicles and exosomes. Biochim. Biophys. Acta Mol. Cell Res..

[24] Donoso-Quezada J., Ayala-Mar S., González-Valdez J. (2021). The role of lipids in exosome biology and intercellular communication: Function, analytics and applications. Traffic.

[25] D’Anca M., Fenoglio C., Buccellato F.R., Visconte C., Galimberti D., Scarpini E. (2021). Extracellular Vesicles in Multiple Sclerosis: Role in the Pathogenesis and Potential Usefulness as Biomarkers and Therapeutic Tools. Cells.

[26] Elzanowska J., Semira C., Costa-Silva B. (2021). DNA in extracellular vesicles: Biological and clinical aspects. Mol. Oncol..

[27] Zhao G., Zhou A., Li X., Zhu S., Wang Y., Zhang S., Li P. (2021). The Significance of Exosomal RNAs in the Development, Diagnosis, and Treatment of Gastric Cancer. Genes.

[28] Paskeh M.D.A., Entezari M., Mirzaei S., Zabolian A., Saleki H., Naghdi M.J., Sabet S., Khoshbakht M.A., Hashemi M., Hushmandi K. (2022). Emerging role of exosomes in cancer progression and tumor microenvironment remodeling. J. Hematol. Oncol..

[29] Zhong Y., Li H., Li P., Chen Y., Zhang M., Yuan Z., Zhang Y., Xu Z., Luo G., Fang Y. (2021). Exosomes: A New Pathway for Cancer Drug Resistance. Front. Oncol..

[30] Sung H., Ferlay J., Siegel R.L., Laversanne M., Soerjomataram I., Jemal A., Bray F. (2021). Global Cancer Statistics 2020: GLOBOCAN Estimates of Incidence and Mortality Worldwide for 36 Cancers in 185 Countries. CA Cancer J. Clin..

[31] Qu Z., Wu J., Wu J., Ji A., Qiang G., Jiang Y., Jiang C., Ding Y. (2017). Exosomal miR-665 as a novel minimally invasive biomarker for hepatocellular carcinoma diagnosis and prognosis. Oncotarget.

[32] Cho H.J., Eun J.W., Baek G.O., Seo C.W., Ahn H.R., Kim S.S., Cho S.W., Cheong J.Y. (2020). Serum Exosomal MicroRNA, miR-10b-5p, as a Potential Diagnostic Biomarker for Early-Stage Hepatocellular Carcinoma. J. Clin. Med..

[33] Xu Y., Lai Y., Cao L., Li Y., Chen G., Chen L., Weng H., Chen T., Wang L., Ye Y. (2021). Human. umbilical cord mesenchymal stem cells-derived exosomal microRNA-451a represses epithelial-mesenchymal transition of hepatocellular carcinoma cells by inhibiting ADAM10. RNA Biol..

[34] Fründt T., Krause L., Hussey E., Steinbach B., Köhler D., von Felden J., Schulze K., Lohse A.W., Wege H., Schwarzenbach H. (2021). Diagnostic and Prognostic Value of miR-16, miR-146a, miR-192 and miR-221 in Exosomes of Hepatocellular Carcinoma and Liver Cirrhosis Patients. Cancers.

[35] Zhou Y., Ren H., Dai B., Li J., Shang L., Huang J., Shi X. (2018). Hepatocellular carcinoma-derived exosomal miRNA-21 contributes to tumor progression by converting hepatocyte stellate cells to cancer-associated fibroblasts. J. Exp. Clin. Cancer Res..

[36] Xue X., Wang X., Zhao Y., Hu R., Qin L. (2018). Exosomal miR-93 promotes proliferation and invasion in hepatocellular carcinoma by directly inhibiting TIMP2/TP53INP1/CDKN1A. Biochem. Biophys. Res. Commun..

[37] Hu Z., Chen J., Zhao Y., Yan C., Wang Y., Zhu J., Li L. (2022). Exosomal miR-452-5p Induce M2 Macrophage Polarization to Accelerate Hepatocellular Carcinoma Progression by Targeting TIMP3. J. Immunol. Res..

[38] Liu H., Chen W., Zhi X., Chen E.J., Wei T., Zhang J., Shen J., Hu L.Q., Zhao B., Feng X.H. (2018). Tumor-derived exosomes promote tumor self-seeding in hepatocellular carcinoma by transferring miRNA-25-5p to enhance cell motility. Oncogene.

[39] Fang J.H., Zhang Z.J., Shang L.R., Luo Y.W., Lin Y.F., Yuan Y., Zhuang S.M. (2018). Hepatoma cell-secreted exosomal microRNA-103 increases vascular permeability and promotes metastasis by targeting junction proteins. Hepatology.

[40] Yokota Y., Noda T., Okumura Y., Kobayashi S., Iwagami Y., Yamada D., Tomimaru Y., Akita H., Gotoh K., Takeda Y. (2021). Serum exosomal miR-638 is a prognostic marker of HCC via downregulation of VE-cadherin and ZO-1 of endothelial cells. Cancer Sci..

[41] Yang B., Feng X., Liu H., Tong R., Wu J., Li C., Yu H., Chen Y., Cheng Q., Chen J. (2020). High-metastatic cancer cells derived exosomal miR92a-3p promotes epithelial-mesenchymal transition and metastasis of low-metastatic cancer cells by regulating PTEN/Akt pathway in hepatocellular carcinoma. Oncogene.

[42] Wang Q., Wang G., Niu L., Zhao S., Li J., Zhang Z., Jiang H., Zhang Q., Wang H., Sun P. (2021). Exosomal MiR-1290 Promotes Angiogenesis of Hepatocellular Carcinoma via Targeting SMEK1. J. Oncol..

[43] Fu X., Liu M., Qu S., Ma J., Zhang Y., Shi T., Wen H., Yang Y., Wang S., Wang J. (2018). Exosomal microRNA-32-5p induces multidrug resistance in hepatocellular carcinoma via the PI3K/Akt pathway. J. Exp. Clin. Cancer Res..

[44] Yu Y., Min Z., Zhou Z., Mao L., Tao R., Yan L., Song H. (2019). Hypoxia-induced exosomes promote hepatocellular carcinoma proliferation and metastasis via miR-1273f transfer. Exp. Cell Res..

[45] Hu Y., Zai H., Jiang W., Yao Y., Ou Z., Zhu Q. (2021). miR-126 in Extracellular Vesicles Derived from Hepatoblastoma Cells Promotes the Tumorigenesis of Hepatoblastoma through Inducing the Differentiation of BMSCs into Cancer Stem Cells. J. Immunol. Res..

[46] Tian X.P., Wang C.Y., Jin X.H., Li M., Wang F.W., Huang W.J., Yun J.P., Xu R.H., Cai Q.Q., Xie D. (2019). Acidic Microenvironment Up-Regulates Exosomal miR-21 and miR-10b in Early-Stage Hepatocellular Carcinoma to Promote Cancer Cell Proliferation and Metastasis. Theranostics.

[47] Liu J., Fan L., Yu H., Zhang J., He Y., Feng D., Wang F., Li X., Liu Q., Li Y. (2019). Endoplasmic Reticulum Stress Causes Liver Cancer Cells to Release Exosomal miR-23a-3p and Up-regulate Programmed Death Ligand 1 Expression in Macrophages. Hepatology.

[48] Gai X., Tang B., Liu F., Wu Y., Wang F., Jing Y., Huang F., Jin D., Wang L., Zhang H. (2019). mTOR/miR-145-regulated exosomal GOLM1 promotes hepatocellular carcinoma through augmented GSK-3β/MMPs. J. Genet. Genom..

[49] Tian B., Zhou L., Wang J., Yang P. (2021). miR-660-5p-loaded M2 macrophages-derived exosomes augment hepatocellular carcinoma development through regulating KLF3. Int. Immunopharmacol..

[50] Yugawa K., Yoshizumi T., Mano Y., Itoh S., Harada N., Ikegami T., Kohashi K., Oda Y., Mori M. (2021). Cancer-associated fibroblasts promote hepatocellular carcinoma progression through downregulation of exosomal miR-150-3p. Eur. J. Surg. Oncol..

[51] Zhang Z., Li X., Sun W., Yue S., Yang J., Li J., Ma B., Wang J., Yang X., Pu M. (2017). Loss of exosomal miR-320a from cancer-associated fibroblasts contributes to HCC proliferation and metastasis. Cancer Lett..

[52] Fang T., Lv H., Lv G., Li T., Wang C., Han Q., Yu L., Su B., Guo L., Huang S. (2018). Tumor-derived exosomal miR-1247-3p induces cancer-associated fibroblast activation to foster lung metastasis of liver cancer. Nat. Commun..

[53] Qi Y., Wang H., Zhang Q., Liu Z., Wang T., Wu Z., Wu W. (2022). CAF-Released Exosomal miR-20a-5p Facilitates HCC Progression via the LIMA1-Mediated β-Catenin Pathway. Cells.

[54] Wang G., Zhao W., Wang H., Qiu G., Jiang Z., Wei G., Li X. (2019). Exosomal MiR-744 Inhibits Proliferation and Sorafenib Chemoresistance in Hepatocellular Carcinoma by Targeting PAX2. Med. Sci. Monit..

[55] Lou G., Chen L., Xia C., Wang W., Qi J., Li A., Zhao L., Chen Z., Zheng M., Liu Y. (2020). MiR-199a-modified exosomes from adipose tissue-derived mesenchymal stem cells improve hepatocellular carcinoma chemosensitivity through mTOR pathway. J. Exp. Clin. Cancer Res..

[56] Lou G., Song X., Yang F., Wu S., Wang J., Chen Z., Liu Y. (2015). Exosomes derived from miR-122-modified adipose tissue-derived MSCs increase chemosensitivity of hepatocellular carcinoma. J. Hematol. Oncol..

[57] De la Cruz-Ojeda P., Schmid T., Boix L., Moreno M., Sapena V., Praena-Fernández J.M., Castell F.J., Falcón-Pérez J.M., Reig M., Brüne B. (2022). miR-200c-3p, miR-222-5p, and miR-512-3p Constitute a Biomarker Signature of Sorafenib Effectiveness in Advanced Hepatocellular Carcinoma. Cells.

[58] Tian S., Zhou X., Zhang M., Cui L., Li B., Liu Y., Su R., Sun K., Hu Y., Yang F. (2022). Mesenchymal stem cell-derived exosomes protect against liver fibrosis via delivering miR-148a to target KLF6/STAT3 pathway in macrophages. Stem Cell Res. Ther..

[59] Ma Y.S., Liu J.B., Lin L., Zhang H., Wu J.J., Shi Y., Jia C.Y., Zhang D.D., Yu F., Wang H.M. (2021). Exosomal microRNA-15a from mesenchymal stem cells impedes hepatocellular carcinoma progression via downregulation of SALL4. Cell Death Discov..

[60] Sun Q., Zhang X., Tan Z., Gu H., Ding S., Ji Y. (2021). Bone marrow mesenchymal stem cells-secreted exosomal microRNA-205-5p exerts inhibitory effect on the progression of liver cancer through regulating CDKL3. Pathol. Res. Pract..

[61] Liang G., Kan S., Zhu Y., Feng S., Feng W., Gao S. (2018). Engineered exosome-mediated delivery of functionally active miR-26a and its enhanced suppression effect in HepG2 cells. Int. J. Nanomed..

[62] Zhang X., Chen F., Huang P., Wang X., Zhou K., Zhou C., Yu L., Peng Y., Fan J., Zhou J. (2022). Exosome-depleted MiR-148a-3p derived from Hepatic Stellate Cells Promotes Tumor Progression via ITGA5/PI3K/Akt Axis in Hepatocellular Carcinoma. Int. J. Biol. Sci..

[63] Wang F., Li L., Piontek K., Sakaguchi M., Selaru F.M. (2018). Exosome miR-335 as a novel therapeutic strategy in hepatocellular carcinoma. Hepatology.

[64] Sun C., Shi C., Duan X., Zhang Y., Wang B. (2022). Exosomal microRNA-618 derived from mesenchymal stem cells attenuate the progression of hepatic fibrosis by targeting Smad4. Bioengineered.

[65] Hao X., Xin R., Dong W. (2020). Decreased serum exosomal miR-320a expression is an unfavorable prognostic factor in patients with hepatocellular carcinoma. J. Int. Med. Res..

[66] Deng P., Li M., Wu Y. (2022). The Predictive Efficacy of Serum Exosomal microRNA-122 and microRNA-148a for Hepatocellular Carcinoma Based on Smart Healthcare. J. Healthc. Eng..

[67] Tang J., Li Y., Liu K., Zhu Q., Yang W.H., Xiong L.K., Guo D.L. (2018). Exosomal miR-9-3p suppresses HBGF-5 expression and is a functional biomarker in hepatocellular carcinoma. Minerva Med..

[68] Li W., Ding X., Wang S., Xu L., Yin T., Han S., Geng J., Sun W. (2020). Downregulation of serum exosomal miR-320d predicts poor prognosis in hepatocellular carcinoma. J. Clin. Lab. Anal..

[69] Zhao S., Li J., Zhang G., Wang Q., Wu C., Zhang Q., Wang H., Sun P., Xiang R., Yang S. (2019). Exosomal miR-451a Functions as a Tumor Suppressor in Hepatocellular Carcinoma by Targeting LPIN1. Cell Physiol. Biochem..

[70] Xiong L., Zhen S., Yu Q., Gong Z. (2017). HCV-E2 inhibits hepatocellular carcinoma metastasis by stimulating mast cells to secrete exosomal shuttle microRNAs. Oncol. Lett..

[71] Ladabaum U., Dominitz J.A., Kahi C., Schoen R.E. (2020). Strategies for Colorectal Cancer Screening. Gastroenterology.

[72] Han B., Zhang H., Tian R., Liu H., Wang Z., Wang Z., Tian J., Cui Y., Ren S., Zuo X. (2022). Exosomal EPHA2 derived from highly metastatic breast cancer cells promotes angiogenesis by activating the AMPK signaling pathway through Ephrin A1-EPHA2 forward signaling. Theranostics.

[73] Liu H., Liu Y., Sun P., Leng K., Xu Y., Mei L., Han P., Zhang B., Yao K., Li C. (2020). Colorectal cancer-derived exosomal miR-106b-3p promotes metastasis by down-regulating DLC-1 expression. Clin. Sci. (Lond.).

[74] Li W., Chang J., Wang S., Liu X., Peng J., Huang D., Sun M., Chen Z., Zhang W., Guo W. (2015). miRNA-99b-5p suppresses liver metastasis of colorectal cancer by down-regulating mTOR. Oncotarget.

[75] Jiang H., Liu J., Chen Y., Ma C., Li B., Hao T. (2016). Up-regulation of mir-10b predicate advanced clinicopathological features and liver metastasis in colorectal cancer. Cancer Med..

[76] Yan S., Jiang Y., Liang C., Cheng M., Jin C., Duan Q., Xu D., Yang L., Zhang X., Ren B. (2018). Exosomal miR-6803-5p as potential diagnostic and prognostic marker in colorectal cancer. J. Cell Biochem..

[77] Bai J., Zhang X., Shi D., Xiang Z., Wang S., Yang C., Liu Q., Huang S., Fang Y., Zhang W. (2021). Exosomal miR-128-3p Promotes Epithelial-to-Mesenchymal Transition in Colorectal Cancer Cells by Targeting FOXO4 via TGF-β/SMAD and JAK/STAT3 Signaling. Front. Cell Dev. Biol..

[78] Zeng Z., Li Y., Pan Y., Lan X., Song F., Sun J., Zhou K., Liu X., Ren X., Wang F. (2018). Cancer-derived exosomal miR-25-3p promotes pre-metastatic niche formation by inducing vascular permeability and angiogenesis. Nat. Commun..

[79] Dai W., Zhou J., Wang H., Zhang M., Yang X., Song W. (2020). miR-424-5p promotes the proliferation and metastasis of colorectal cancer by directly targeting SCN4B. Pathol. Res. Pract..

[80] Sun L.H., Tian D., Yang Z.C., Li J.L. (2020). Exosomal miR-21 promotes proliferation, invasion and therapy resistance of colon adenocarcinoma cells through its target PDCD4. Sci. Rep..

[81] Uratani R., Toiyama Y., Kitajima T., Kawamura M., Hiro J., Kobayashi M., Tanaka K., Inoue Y., Mohri Y., Mori T. (2016). Diagnostic Potential of Cell-Free and Exosomal MicroRNAs in the Identification of Patients with High-Risk Colorectal Adenomas. PLoS ONE.

[82] Wu F., Yang J., Shang G., Zhang Z., Niu S., Liu Y., Liu H., Jing J., Fang Y. (2022). Exosomal miR-224-5p from Colorectal Cancer Cells Promotes Malignant Transformation of Human Normal Colon Epithelial Cells by Promoting Cell Proliferation through Downregulation of CMTM4. Oxidative Med. Cell. Longev..

[83] Dohmen J., Semaan A., Kobilay M., Zaleski M., Branchi V., Schlierf A., Hettwer K., Uhlig S., Hartmann G., Kalff J.C. (2022). Diagnostic Potential of Exosomal microRNAs in Colorectal Cancer. Diagnostics.

[84] Yang C., Dou R., Wei C., Liu K., Shi D., Zhang C., Liu Q., Wang S., Xiong B. (2021). Tumor-derived exosomal microRNA-106b-5p activates EMT-cancer cell and M2-subtype TAM interaction to facilitate CRC metastasis. Mol. Ther..

[85] Ge L., Zhou F., Nie J., Wang X., Zhao Q. (2021). Hypoxic colorectal cancer-secreted exosomes deliver miR-210-3p to normoxic tumor cells to elicit a protumoral effect. Exp. Biol. Med..

[86] Qu M., Li J., Hong Z., Jia F., He Y., Yuan L. (2022). The role of human umbilical cord mesenchymal stem cells-derived exosomal microRNA-431-5p in survival and prognosis of colorectal cancer patients. Mutagenesis.

[87] Zhao S., Mi Y., Guan B., Zheng B., Wei P., Gu Y., Zhang Z., Cai S., Xu Y., Li X. (2020). Tumor-derived exosomal miR-934 induces macrophage M2 polarization to promote liver metastasis of colorectal cancer. J. Hematol. Oncol..

[88] Shang A., Wang X., Gu C., Liu W., Sun J., Zeng B., Chen C., Ji P., Wu J., Quan W. (2020). Exosomal miR-183-5p promotes angiogenesis in colorectal cancer by regulation of FOXO1. Aging.

[89] He Q., Ye A., Ye W., Liao X., Qin G., Xu Y., Yin Y., Luo H., Yi M., Xian L. (2021). Cancer-secreted exosomal miR-21-5p induces angiogenesis and vascular permeability by targeting KRIT1. Cell Death Dis..

[90] Hu H.Y., Yu C.H., Zhang H.H., Zhang S.Z., Yu W.Y., Yang Y., Chen Q. (2019). Exosomal miR-1229 derived from colorectal cancer cells promotes angiogenesis by targeting HIPK2. Int. J. Biol. Macromol..

[91] Dou R., Liu K., Yang C., Zheng J., Shi D., Lin X., Wei C., Zhang C., Fang Y., Huang S. (2021). EMT-cancer cells-derived exosomal miR-27b-3p promotes circulating tumour cells-mediated metastasis by modulating vascular permeability in colorectal cancer. Clin. Transl. Med..

[92] Chen X., Liu J., Zhang Q., Liu B., Cheng Y., Zhang Y., Sun Y., Ge H., Liu Y. (2020). Exosome-mediated transfer of miR-93-5p from cancer-associated fibroblasts confer radioresistance in colorectal cancer cells by downregulating FOXA1 and upregulating TGFB3. J. Exp. Clin. Cancer Res..

[93] Chen X., Liu Y., Zhang Q., Liu B., Cheng Y., Zhang Y., Sun Y., Liu J. (2021). Exosomal miR-590-3p derived from cancer-associated fibroblasts confers radioresistance in colorectal cancer. Mol. Ther. Nucleic Acids.

[94] Zhang Y., Yin C., Wei C., Xia S., Qiao Z., Zhang X.W., Yu B., Zhou J., Wang R. (2022). Exosomal miR-625-3p secreted by cancer-associated fibroblasts in colorectal cancer promotes EMT and chemotherapeutic resistance by blocking the CELF2/WWOX pathway. Pharm. Res..

[95] Dai G., Yao X., Zhang Y., Gu J., Geng Y., Xue F., Zhang J. (2018). Colorectal cancer cell-derived exosomes containing miR-10b regulate fibroblast cells via the PI3K/Akt pathway. Bull. Cancer.

[96] Zhang Y., Wang S., Lai Q., Fang Y., Wu C., Liu Y., Li Q., Wang X., Gu C., Chen J. (2020). Cancer-associated fibroblasts-derived exosomal miR-17-5p promotes colorectal cancer aggressive phenotype by initiating a RUNX3/MYC/TGF-β1 positive feedback loop. Cancer Lett..

[97] Wang D., Wang X., Song Y., Si M., Sun Y., Liu X., Cui S., Qu X., Yu X. (2022). Exosomal miR-146a-5p and miR-155-5p promote CXCL12/CXCR7-induced metastasis of colorectal cancer by crosstalk with cancer-associated fibroblasts. Cell Death Dis..

[98] Han J., Sun W., Liu R., Zhou Z., Zhang H., Chen X., Ba Y. (2020). Plasma Exosomal miRNA Expression Profile as Oxaliplatin-Based Chemoresistant Biomarkers in Colorectal Adenocarcinoma. Front. Oncol..

[99] Jin G., Liu Y., Zhang J., Bian Z., Yao S., Fei B., Zhou L., Yin Y., Huang Z. (2019). A panel of serum exosomal microRNAs as predictive markers for chemoresistance in advanced colorectal cancer. Cancer Chemother. Pharm..

[100] Xu Y., Zhu M. (2020). Novel exosomal miR-46146 transfer oxaliplatin chemoresistance in colorectal cancer. Clin. Transl. Oncol..

[101] Xiao Z., Liu Y., Li Q., Liu Q., Liu Y., Luo Y., Wei S. (2021). EVs delivery of miR-1915-3p improves the chemotherapeutic efficacy of oxaliplatin in colorectal cancer. Cancer Chemother. Pharm..

[102] Hsu H.H., Kuo W.W., Shih H.N., Cheng S.F., Yang C.K., Chen M.C., Tu C.C., Viswanadha V.P., Liao P.H., Huang C.Y. (2019). FOXC1 Regulation of miR-31-5p Confers Oxaliplatin Resistance by Targeting LATS2 in Colorectal Cancer. Cancers.

[103] Yan S., Ren X., Yang J., Wang J., Zhang Q., Xu D. (2020). Exosomal miR-548c-5p Regulates Colorectal Cancer Cell Growth and Invasion Through HIF1A/CDC42 Axis. OncoTargets Ther..

[104] Hosseini M., Baghaei K., Hajivalili M., Zali M.R., Ebtekar M., Amani D. (2022). The anti-tumor effects of CT-26 derived exosomes enriched by MicroRNA-34a on murine model of colorectal cancer. Life Sci..

[105] Jahangiri B., Khalaj-Kondori M., Asadollahi E., Purrafee Dizaj L., Sadeghizadeh M. (2022). MSC-Derived exosomes suppress colorectal cancer cell proliferation and metastasis via miR-100/mTOR/miR-143 pathway. Int. J. Pharm..

[106] Zhang Y., Liu W.S., Zhang X.Y., Tong H.X., Yang H., Liu W.F., Fan J., Zhou J., Hu J. (2022). Low expression of exosomal miR-150 predicts poor prognosis in colorectal cancer patients after surgical resections. Carcinogenesis.

[107] Peng Z.Y., Gu R.H., Yan B. (2019). Downregulation of exosome-encapsulated miR-548c-5p is associated with poor prognosis in colorectal cancer. J. Cell. Biochem..

[108] Mokutani Y., Uemura M., Munakata K., Okuzaki D., Haraguchi N., Takahashi H., Nishimura J., Hata T., Murata K., Takemasa I. (2016). Down-Regulation of microRNA-132 is Associated with Poor Prognosis of Colorectal Cancer. Ann. Surg. Oncol..

[109] Liu D., Chen C., Cui M., Zhang H. (2021). miR-140-3p inhibits colorectal cancer progression and its liver metastasis by targeting BCL9 and BCL2. Cancer Med..

[110] Zhang N., Zhang P.P., Huang J.J., Wang Z.Y., Zhang Z.H., Yuan J.Z., Ma E.M., Liu X., Bai J. (2020). Reduced serum exosomal miR-874 expression predicts poor prognosis in colorectal cancer. Eur. Rev. Med. Pharm. Sci..

[111] Li T., Wan Y., Su Z., Li J., Han M., Zhou C. (2021). Mesenchymal Stem Cell-Derived Exosomal microRNA-3940-5p Inhibits Colorectal Cancer Metastasis by Targeting Integrin α6. Dig. Dis. Sci..

[112] Tong F., Ying Y., Pan H., Zhao W., Li H., Zhan X. (2018). MicroRNA-466 (miR-466) functions as a tumor suppressor and prognostic factor in colorectal cancer (CRC). Bosn. J. Basic Med. Sci..

[113] Xuan B., Wang Y. (2023). Exosome-Transmitted miR-506-3p Inhibits Colorectal Cancer Cell Malignancy via Regulating GSTP1. Appl. Biochem. Biotechnol..

[114] Zou S.L., Chen Y.L., Ge Z.Z., Qu Y.Y., Cao Y., Kang Z.X. (2019). Downregulation of serum exosomal miR-150-5p is associated with poor prognosis in patients with colorectal cancer. Cancer Biomark..

[115] Miller K.D., Nogueira L., Devasia T., Mariotto A.B., Yabroff K.R., Jemal A., Kramer J., Siegel R.L. (2022). Cancer treatment and survivorship statistics, 2022. CA Cancer J. Clin..

[116] Sharifi Z., Talkhabi M., Taleahmad S. (2022). Identification of potential microRNA diagnostic panels and uncovering regulatory mechanisms in breast cancer pathogenesis. Sci. Rep..

[117] Asadirad A., Khodadadi A., Talaiezadeh A., Shohan M., Rashno M., Joudaki N. (2022). Evaluation of miRNA-21-5p and miRNA-10b-5p levels in serum-derived exosomes of breast cancer patients in different grades. Mol. Cell. Probes.

[118] Li D., Wang J., Ma L.J., Yang H.B., Jing J.F., Jia M.M., Zhang X.J., Guo F., Gao J.N. (2020). Identification of serum exosomal miR-148a as a novel prognostic biomarker for breast cancer. Eur. Rev. Med. Pharm. Sci..

[119] Lv S., Wang Y., Xu W., Dong X. (2020). Serum Exosomal miR-17-5p as a Promising Biomarker Diagnostic Biomarker for Breast Cancer. Clin. Lab..

[120] Sueta A., Yamamoto Y., Tomiguchi M., Takeshita T., Yamamoto-Ibusuki M., Iwase H. (2017). Differential expression of exosomal miRNAs between breast cancer patients with and without recurrence. Oncotarget.

[121] Shen S., Song Y., Zhao B., Xu Y., Ren X., Zhou Y., Sun Q. (2021). Cancer-derived exosomal miR-7641 promotes breast cancer progression and metastasis. Cell Commun. Signal..

[122] Wang X., Qian T., Bao S., Zhao H., Chen H., Xing Z., Li Y., Zhang M., Meng X., Wang C. (2021). Circulating exosomal miR-363-5p inhibits lymph node metastasis by downregulating PDGFB and serves as a potential noninvasive biomarker for breast cancer. Mol. Oncol..

[123] Liu M., Mo F., Song X., He Y., Yuan Y., Yan J., Yang Y., Huang J., Zhang S. (2021). Exosomal hsa-miR-21-5p is a biomarker for breast cancer diagnosis. PeerJ.

[124] Xin Y., Wang X., Meng K., Ni C., Lv Z., Guan D. (2020). Identification of exosomal miR-455-5p and miR-1255a as therapeutic targets for breast cancer. Biosci. Rep..

[125] Wang B., Mao J.H., Wang B.Y., Wang L.X., Wen H.Y., Xu L.J., Fu J.X., Yang H. (2020). Exosomal miR-1910-3p promotes proliferation, metastasis, and autophagy of breast cancer cells by targeting MTMR3 and activating the NF-κB signaling pathway. Cancer Lett..

[126] Wang J., Zhang Q., Wang D., Yang S., Zhou S., Xu H., Zhang H., Zhong S., Feng J. (2020). Microenvironment-induced TIMP2 loss by cancer-secreted exosomal miR-4443 promotes liver metastasis of breast cancer. J. Cell. Physiol..

[127] Huang X., Lai S., Qu F., Li Z., Fu X., Li Q., Zhong X., Wang C., Li H. (2022). CCL18 promotes breast cancer progression by exosomal miR-760 activation of ARF6/Src/PI3K/Akt pathway. Mol. Ther. Oncolytics.

[128] O’Brien K., Lowry M.C., Corcoran C., Martinez V.G., Daly M., Rani S., Gallagher W.M., Radomski M.W., MacLeod R.A., O’Driscoll L. (2015). miR-134 in extracellular vesicles reduces triple-negative breast cancer aggression and increases drug sensitivity. Oncotarget.

[129] Li X.J., Ren Z.J., Tang J.H., Yu Q. (2017). Exosomal MicroRNA MiR-1246 Promotes Cell Proliferation, Invasion and Drug Resistance by Targeting CCNG2 in Breast Cancer. Cell. Physiol. Biochem..

[130] Zhu Y., Dou H., Liu Y., Yu P., Li F., Wang Y., Xiao M. (2022). Breast Cancer Exosome-Derived miR-425-5p Induces Cancer-Associated Fibroblast-Like Properties in Human Mammary Fibroblasts by TGFβ1/ROS Signaling Pathway. Oxidative Med. Cell. Longev..

[131] Liang Z., Liu L., Gao R., Che C., Yang G. (2022). Downregulation of exosomal miR-7-5p promotes breast cancer migration and invasion by targeting RYK and participating in the atypical WNT signalling pathway. Cell. Mol. Biol. Lett..

[132] Du L., Tao X., Shen X. (2021). Human umbilical cord mesenchymal stem cell-derived exosomes inhibit migration and invasion of breast cancer cells via miR-21-5p/ZNF367 pathway. Breast Cancer.

[133] Ozawa P.M.M., Alkhilaiwi F., Cavalli I.J., Malheiros D., de Souza Fonseca Ribeiro E.M., Cavalli L.R. (2018). Extracellular vesicles from triple-negative breast cancer cells promote proliferation and drug resistance in non-tumorigenic breast cells. Breast Cancer Res. Treat..

[134] Huang S., Fan P., Zhang C., Xie J., Gu X., Lei S., Chen Z., Huang Z. (2021). Exosomal microRNA-503-3p derived from macrophages represses glycolysis and promotes mitochondrial oxidative phosphorylation in breast cancer cells by elevating DACT2. Cell Death Discov..

[135] Scognamiglio I., Cocca L., Puoti I., Palma F., Ingenito F., Quintavalle C., Affinito A., Roscigno G., Nuzzo S., Chianese R.V. (2022). Exosomal microRNAs synergistically trigger stromal fibroblasts in breast cancer. Mol. Ther. Nucleic Acids.

[136] Chen B., Sang Y., Song X., Zhang D., Wang L., Zhao W., Liang Y., Zhang N., Yang Q. (2021). Exosomal miR-500a-5p derived from cancer-associated fibroblasts promotes breast cancer cell proliferation and metastasis through targeting USP28. Theranostics.

[137] Yan Z., Sheng Z., Zheng Y., Feng R., Xiao Q., Shi L., Li H., Yin C., Luo H., Hao C. (2021). Cancer-associated fibroblast-derived exosomal miR-18b promotes breast cancer invasion and metastasis by regulating TCEAL7. Cell Death Dis..

[138] Pakravan K., Mossahebi-Mohammadi M., Ghazimoradi M.H., Cho W.C., Sadeghizadeh M., Babashah S. (2022). Monocytes educated by cancer-associated fibroblasts secrete exosomal miR-181a to activate AKT signaling in breast cancer cells. J. Transl. Med..

[139] Ni Q., Stevic I., Pan C., Müller V., Oliveira-Ferrer L., Pantel K., Schwarzenbach H. (2018). Different signatures of miR-16, miR-30b and miR-93 in exosomes from breast cancer and DCIS patients. Sci. Rep..

[140] Eichelser C., Stückrath I., Müller V., Milde-Langosch K., Wikman H., Pantel K., Schwarzenbach H. (2014). Increased serum levels of circulating exosomal microRNA-373 in receptor-negative breast cancer patients. Oncotarget.

[141] Curtaz C.J., Reifschläger L., Strähle L., Feldheim J., Feldheim J.J., Schmitt C., Kiesel M., Herbert S.L., Wöckel A., Meybohm P. (2022). Analysis of microRNAs in Exosomes of Breast Cancer Patients in Search of Molecular Prognostic Factors in Brain Metastases. Int. J. Mol. Sci..

[142] Yang Q., Zhao S., Shi Z., Cao L., Liu J., Pan T., Zhou D., Zhang J. (2021). Chemotherapy-elicited exosomal miR-378a-3p and miR-378d promote breast cancer stemness and chemoresistance via the activation of EZH2/STAT3 signaling. J. Exp. Clin. Cancer Res..

[143] Zhang Z., Zhang L., Yu G., Sun Z., Wang T., Tian X., Duan X., Zhang C. (2020). Exosomal miR-1246 and miR-155 as predictive and prognostic biomarkers for trastuzumab-based therapy resistance in HER2-positive breast cancer. Cancer Chemother. Pharm..

[144] Shen M., Dong C., Ruan X., Yan W., Cao M., Pizzo D., Wu X., Yang L., Liu L., Ren X. (2019). Chemotherapy-Induced Extracellular Vesicle miRNAs Promote Breast Cancer Stemness by Targeting ONECUT2. Cancer Res..

[145] Han M., Hu J., Lu P., Cao H., Yu C., Li X., Qian X., Yang X., Yang Y., Han N. (2020). Exosome-transmitted miR-567 reverses trastuzumab resistance by inhibiting ATG5 in breast cancer. Cell Death Dis..

[146] Koh M.Z., Ho W.Y., Yeap S.K., Ali N.M., Yong C.Y., Boo L., Alitheen N.B. (2022). Exosomal-microRNA transcriptome profiling of Parental and CSC-like MDA-MB-231 cells in response to cisplatin treatment. Pathol. Res. Pract..

[147] Rodríguez-Martínez A., de Miguel-Pérez D., Ortega F.G., García-Puche J.L., Robles-Fernández I., Exposito J., Martorell-Marugan J., Carmona-Sáez P., Garrido-Navas M.D.C., Rolfo C. (2019). Exosomal miRNA profile as complementary tool in the diagnostic and prediction of treatment response in localized breast cancer under neoadjuvant chemotherapy. Breast Cancer Res..

[148] Li S., Zhang M., Xu F., Wang Y., Leng D. (2021). Detection significance of miR-3662, miR-146a, and miR-1290 in serum exosomes of breast cancer patients. J. Cancer Res. Ther..

[149] Zhao Y., Jin L.J., Zhang X.Y. (2021). Exosomal miRNA-205 promotes breast cancer chemoresistance and tumorigenesis through E2F1. Aging.

[150] Zhang Y., Lai X., Yue Q., Cao F., Zhang Y., Sun Y., Tian J., Lu Y., He L., Bai J. (2022). Bone marrow mesenchymal stem cells-derived exosomal microRNA-16-5p restrains epithelial-mesenchymal transition in breast cancer cells via EPHA1/NF-κB signaling axis. Genomics.

[151] Yang Z., Xu B., Wu S., Yang W., Luo R., Geng S., Xin Z., Jin W., Shen X., Gu X. (2022). Exosomal microRNA-551b-3p from bone marrow-derived mesenchymal stromal cells inhibits breast cancer progression via regulating TRIM31/Akt signaling. Hum. Cell.

[152] Gu Z., Yin H., Zhang H., Zhang H., Liu X., Zeng X., Zheng X. (2022). Optimization of a method for the clinical detection of serum exosomal miR-940 as a potential biomarker of breast cancer. Front. Oncol..

[153] Yang C., Zhang G., Zhang Y., Zhang S., Li J., Liu Y. (2021). Exosome miR-134-5p restrains breast cancer progression via regulating PI3K/AKT pathway by targeting ARHGAP1. J. Obstet. Gynaecol. Res..

[154] Khazaei-Poul Y., Shojaei S., Koochaki A., Ghanbarian H., Mohammadi-Yeganeh S. (2021). Evaluating the influence of Human. Umbilical Cord. Mesenchymal Stem Cells-derived exosomes loaded with miR-3182 on metastatic performance of Triple Negative Breast Cancer cells. Life Sci..

[155] Jain G., Das P., Ranjan P., Valderrama F., Cieza-Borrella C. (2023). Urinary extracellular vesicles miRNA-A new era of prostate cancer biomarkers. Front. Genet..

[156] Bhagirath D., Yang T.L., Bucay N., Sekhon K., Majid S., Shahryari V., Dahiya R., Tanaka Y., Saini S. (2018). microRNA-1246 Is an Exosomal Biomarker for Aggressive Prostate Cancer. Cancer Res..

[157] Huang X., Yuan T., Liang M., Du M., Xia S., Dittmar R., Wang D., See W., Costello B.A., Quevedo F. (2015). Exosomal miR-1290 and miR-375 as prognostic markers in castration-resistant prostate cancer. Eur. Urol..

[158] Endzeliņš E., Berger A., Melne V., Bajo-Santos C., Soboļevska K., Ābols A., Rodriguez M., Šantare D., Rudņickiha A., Lietuvietis V. (2017). Detection of circulating miRNAs: Comparative analysis of extracellular vesicle-incorporated miRNAs and cell-free miRNAs in whole plasma of prostate cancer patients. BMC Cancer.

[159] Li Z., Ma Y.Y., Wang J., Zeng X.F., Li R., Kang W., Hao X.K. (2016). Exosomal microRNA-141 is upregulated in the serum of prostate cancer patients. OncoTargets Ther..

[160] Shin S., Park Y.H., Jung S.H., Jang S.H., Kim M.Y., Lee J.Y., Chung Y.J. (2021). Urinary exosome microRNA signatures as a noninvasive prognostic biomarker for prostate cancer. NPJ Genom. Med..

[161] Zhou C., Chen Y., He X., Zheng Z., Xue D. (2020). Functional Implication of Exosomal miR-217 and miR-23b-3p in the Progression of Prostate Cancer. OncoTargets Ther..

[162] Guan H., Peng R., Fang F., Mao L., Chen Z., Yang S., Dai C., Wu H., Wang C., Feng N. (2020). Tumor-associated macrophages promote prostate cancer progression via exosome-mediated miR-95 transfer. J. Cell. Physiol..

[163] Luedemann C., Reinersmann J.L., Klinger C., Degener S., Dreger N.M., Roth S., Kaufmann M., Savelsbergh A. (2022). Prostate Cancer-Associated miRNAs in Saliva: First Steps to an Easily Accessible and Reliable Screening Tool. Biomolecules.

[164] Zhai T.Y., Dou M., Ma Y.B., Wang H., Liu F., Zhang L.D., Chong T., Wang Z.M., Xue L. (2022). miR-20b-5p is a novel biomarker for detecting prostate cancer. Oncol. Lett..

[165] Rode M.P., Silva A.H., Cisilotto J., Rosolen D., Creczynski-Pasa T.B. (2021). miR-425-5p as an exosomal biomarker for metastatic prostate cancer. Cell. Signal..

[166] Bryant R.J., Pawlowski T., Catto J.W., Marsden G., Vessella R.L., Rhees B., Kuslich C., Visakorpi T., Hamdy F.C. (2012). Changes in circulating microRNA levels associated with prostate cancer. Br. J. Cancer.

[167] Ku A., Fredsøe J., Sørensen K.D., Borre M., Evander M., Laurell T., Lilja H., Ceder Y. (2021). High-Throughput and Automated Acoustic Trapping of Extracellular Vesicles to Identify microRNAs With Diagnostic Potential for Prostate Cancer. Front. Oncol..

[168] Kurniawati I., Liu M.C., Hsieh C.L., Do A.D., Sung S.Y. (2022). Targeting Castration-Resistant Prostate Cancer Using Mesenchymal Stem Cell Exosomes for Therapeutic MicroRNA-let-7c Delivery. Front. Biosci. (Landmark Ed.).

[169] Guo Z., Lu X., Yang F., Qin L., Yang N., Cai P., Han C., Wu J., Wang H. (2022). The Expression of miR-205 in Prostate Carcinoma and the Relationship with Prognosis in Patients. Comput. Math. Methods Med..

[170] Wei Y., Chen Z., Zhang R., Wu B., Lin L., Zhu Q., Ye L., Li T., Li F. (2022). Blood circulating exosomes carrying microRNA-423-5p regulates cell progression in prostate cancer via targeting FRMD3. J. Cancer.

[171] Kim M.Y., Shin H., Moon H.W., Park Y.H., Park J., Lee J.Y. (2021). Urinary exosomal microRNA profiling in intermediate-risk prostate cancer. Sci. Rep..

[172] Albino D., Falcione M., Uboldi V., Temilola D.O., Sandrini G., Merulla J., Civenni G., Kokanovic A., Stürchler A., Shinde D. (2021). Circulating extracellular vesicles release oncogenic miR-424 in experimental models and patients with aggressive prostate cancer. Commun. Biol..

[173] Panigrahi G.K., Ramteke A., Birks D., Abouzeid Ali H.E., Venkataraman S., Agarwal C., Vibhakar R., Miller L.D., Agarwal R., Abd Elmageed Z.Y. (2018). Exosomal microRNA profiling to identify hypoxia-related biomarkers in prostate cancer. Oncotarget.

[174] Dai Y., Gao X. (2021). Inhibition of cancer cell-derived exosomal microRNA-183 suppresses cell growth and metastasis in prostate cancer by upregulating TPM1. Cancer Cell Int..

[175] Sánchez C.A., Andahur E.I., Valenzuela R., Castellón E.A., Fullá J.A., Ramos C.G., Triviño J.C. (2016). Exosomes from bulk and stem cells from human prostate cancer have a differential microRNA content that contributes cooperatively over local and pre-metastatic niche. Oncotarget.

[176] Cho S., Yang H.C., Rhee W.J. (2019). Simultaneous multiplexed detection of exosomal microRNAs and surface proteins for prostate cancer diagnosis. Biosens. Bioelectron..

[177] Bertoli G., Panio A., Cava C., Gallivanone F., Alini M., Strano G., Molfino F., Brioschi L., Viani P., Porro D. (2022). Secreted miR-153 Controls Proliferation and Invasion of Higher Gleason Score Prostate Cancer. Int. J. Mol. Sci..

[178] Foj L., Ferrer F., Serra M., Arévalo A., Gavagnach M., Giménez N., Filella X. (2017). Exosomal and Non-Exosomal Urinary miRNAs in Prostate Cancer Detection and Prognosis. Prostate.

[179] Kim J., Shim J.S., Han B.H., Kim H.J., Park J., Cho I.J., Kang S.G., Kang J.Y., Bong K.W., Choi N. (2021). Hydrogel-based hybridization chain reaction (HCR) for detection of urinary exosomal miRNAs as a diagnostic tool of prostate cancer. Biosens. Bioelectron..

[180] Bryzgunova O.E., Zaripov M.M., Skvortsova T.E., Lekchnov E.A., Grigor’eva A.E., Zaporozhchenko I.A., Morozkin E.S., Ryabchikova E.I., Yurchenko Y.B., Voitsitskiy V.E. (2016). Comparative Study of Extracellular Vesicles from the Urine of Healthy Individuals and Prostate Cancer Patients. PLoS ONE.

[181] Holdmann J., Markert L., Klinger C., Kaufmann M., Schork K., Turewicz M., Eisenacher M., Degener S., Dreger N.M., Roth S. (2022). MicroRNAs from urinary exosomes as alternative biomarkers in the differentiation of benign and malignant prostate diseases. J. Circ. Biomark..

[182] Xu Y., Qin S., An T., Tang Y., Huang Y., Zheng L. (2017). MiR-145 detection in urinary extracellular vesicles increase diagnostic efficiency of prostate cancer based on hydrostatic filtration dialysis method. Prostate.

[183] Matsuzaki K., Fujita K., Tomiyama E., Hatano K., Hayashi Y., Wang C., Ishizuya Y., Yamamoto Y., Hayashi T., Kato T. (2021). MiR-30b-3p and miR-126-3p of urinary extracellular vesicles could be new biomarkers for prostate cancer. Transl. Androl. Urol..

[184] Li Z., Li L.X., Diao Y.J., Wang J., Ye Y., Hao X.K. (2021). Identification of Urinary Exosomal miRNAs for the Non-Invasive Diagnosis of Prostate Cancer. Cancer Manag. Res..

[185] Wani S., Kaul D., Mavuduru R.S., Kakkar N., Bhatia A. (2017). Urinary-exosomal miR-2909: A novel pathognomonic trait of prostate cancer severity. J. Biotechnol..

[186] Ye Y., Li S.L., Ma Y.Y., Diao Y.J., Yang L., Su M.Q., Li Z., Ji Y., Wang J., Lei L. (2017). Exosomal miR-141-3p regulates osteoblast activity to promote the osteoblastic metastasis of prostate cancer. Oncotarget.

[187] Tian G., Hu K., Qiu S., Xie Y., Cao Y., Ni S., Zhang L. (2021). Exosomes derived from PC-3 cells suppress osteoclast differentiation by downregulating miR-148a and blocking the PI3K/AKT/mTOR pathway. Exp. Ther. Med..

[188] Zou Z., Dai R., Deng N., Su W., Liu P. (2021). Exosomal miR-1275 Secreted by Prostate Cancer Cells Modulates Osteoblast Proliferation and Activity by Targeting the SIRT2/RUNX2 Cascade. Cell. Transpl..

[189] Hashimoto K., Ochi H., Sunamura S., Kosaka N., Mabuchi Y., Fukuda T., Yao K., Kanda H., Ae K., Okawa A. (2018). Cancer-secreted hsa-miR-940 induces an osteoblastic phenotype in the bone metastatic microenvironment via targeting ARHGAP1 and FAM134A. Proc. Natl. Acad. Sci. USA.

[190] Duan Y., Tan Z., Yang M., Li J., Liu C., Wang C., Zhang F., Jin Y., Wang Y., Zhu L. (2019). PC-3-Derived Exosomes Inhibit Osteoclast Differentiation by Downregulating miR-214 and Blocking NF-κB Signaling Pathway. Biomed. Res. Int..

[191] Pudova E.A., Kobelyatskaya A.A., Katunina I.V., Snezhkina A.V., Fedorova M.S., Guvatova Z.G., Nyushko K.M., Alekseev B.Y., Pavlov V.S., Savvateeva M.V. (2022). Dynamic Profiling of Exosomal microRNAs in Blood Plasma of Patients with Castration-Resistant Prostate Cancer. Front. Biosci. (Sch. Ed.).

[192] Bhagirath D., Liston M., Akoto T., Lui B., Bensing B.A., Sharma A., Saini S. (2021). Novel, non-invasive markers for detecting therapy induced neuroendocrine differentiation in castration-resistant prostate cancer patients. Sci. Rep..

[193] Corcoran C., Rani S., O’Driscoll L. (2014). miR-34a is an intracellular and exosomal predictive biomarker for response to docetaxel with clinical relevance to prostate cancer progression. Prostate.

[194] Zhang Y., Zhao J., Ding M., Su Y., Cui D., Jiang C., Zhao S., Jia G., Wang X., Ruan Y. (2020). Loss of exosomal miR-146a-5p from cancer-associated fibroblasts after androgen deprivation therapy contributes to prostate cancer metastasis. J. Exp. Clin. Cancer Res..

[195] Gan J., Liu S., Zhang Y., He L., Bai L., Liao R., Zhao J., Guo M., Jiang W., Li J. (2022). MicroRNA-375 is a therapeutic target for castration-resistant prostate cancer through the PTPN4/STAT3 axis. Exp. Mol. Med..

[196] Li J., Yang X., Guan H., Mizokami A., Keller E.T., Xu X., Liu X., Tan J., Hu L., Lu Y. (2016). Exosome-derived microRNAs contribute to prostate cancer chemoresistance. Int. J. Oncol..

[197] Zhang H., Li M., Zhang J., Shen Y., Gui Q. (2021). Exosomal Circ-XIAP Promotes Docetaxel Resistance in Prostate Cancer by Regulating miR-1182/TPD52 Axis. Drug Des. Dev. Ther..

[198] Cao Z., Xu L., Zhao S. (2019). Exosome-derived miR-27a produced by PSC-27 cells contributes to prostate cancer chemoresistance through p53. Biochem. Biophys. Res. Commun..

[199] Cannistraci A., Federici G., Addario A., Di Pace A.L., Grassi L., Muto G., Collura D., Signore M., De Salvo L., Sentinelli S. (2017). C-Met/miR-130b axis as novel mechanism and biomarker for castration resistance state acquisition. Oncogene.

[200] Wang X., Wang X., Zhu Z., Li W., Yu G., Jia Z., Wang X. (2019). Prostate carcinoma cell-derived exosomal MicroRNA-26a modulates the metastasis and tumor growth of prostate carcinoma. Biomed. Pharm..

[201] Shan G., Gu J., Zhou D., Li L., Cheng W., Wang Y., Tang T., Wang X. (2020). Cancer-associated fibroblast-secreted exosomal miR-423-5p promotes chemotherapy resistance in prostate cancer by targeting GREM2 through the TGF-β signaling pathway. Exp. Mol. Med..

[202] Tang Y., Liu J., Li X., Wang W. (2021). Exosomal circRNA HIPK3 knockdown inhibited cell proliferation and metastasis in prostate cancer by regulating miR-212/BMI-1 pathway. J. Biosci..

[203] Li C., Sun Z., Song Y., Zhang Y. (2022). Suppressive function of bone marrow-derived mesenchymal stem cell-derived exosomal microRNA-187 in prostate cancer. Cancer Biol. Ther..

